# Population Genomics Informs Resilience and Vulnerability of Habitat‐Building Coralline Algae

**DOI:** 10.1111/eva.70179

**Published:** 2025-11-17

**Authors:** Tom L. Jenkins, Magnus Axelsson, Angela Gall, Frances Ratcliffe, Charlie D. Ellis, Jamie R. Stevens

**Affiliations:** ^1^ Biosciences, Faculty of Health and Life Sciences University of Exeter Exeter UK; ^2^ Natural England Eastleigh UK; ^3^ Natural Resources Wales Neath UK

**Keywords:** bioinfomatics/phyloinfomatics, conservation genetics, ecological genetics, habitat degradation, population genetics ‐ empirical, wildlife management

## Abstract

Maerl beds, formed by free‐living coralline red algae, are biodiversity‐rich and carbon‐storing habitats of high conservation value but remain understudied at the genomic level. Here, we present the first draft genomes and population genomic analyses for two dominant maerl‐forming species in the north‐east Atlantic, *Phymatolithon calcareum* and *Lithothamnion corallioides*. Using maerl samples genotyped at over 15,000 single nucleotide polymorphisms (SNPs) across England, Wales and additional European sites, we assessed clonal diversity, population structure and potential adaptation to environmental gradients. *P. calcareum* generally showed moderate clonal diversity, though extreme clonality driven by a single genet (multi‐locus lineage) was detected at certain sites. In comparison, *L. corallioides* displayed high clonal diversity, with most maerl samples representing distinct genets, although local dominance of a single genet was occasionally observed. Contrasting clonal dynamics have important implications for resilience, as populations dominated by a few clones may be more sensitive to environmental change. Population structure analyses in both species revealed strong genetic differentiation between sites, consistent with limited dispersal, while genomic associations identified candidate SNPs linked to climate in *P. calcareum* , albeit explaining only a small proportion of the observed genetic variation. Genomic offset analyses suggested that certain populations may require greater shifts in allele frequencies to avoid being maladapted to mid‐century climate scenarios. Together, these findings highlight both genetically diverse and potentially vulnerable maerl populations, some of which fall within existing marine protected areas. Integrating genomic insights with ecological monitoring will help inform conservation and restoration strategies for these irreplaceable, high natural capital value habitats.

## 1 Introduction

Genomic tools have transformed our ability to assess genetic diversity, population structure and local adaptation in natural populations, providing insights that are directly relevant to conservation management (Aguirre‐Liguori et al. 2021; Allendorf et al. 2010; Davey et al. 2011). In particular, conservation genomics underpins the development of Essential Biodiversity Variables for genetic composition (Hoban et al. 2022; Pereira et al. 2013), which aim to provide standardised metrics such as genetic diversity (e.g., heterozygosity), genetic differentiation and effective population size for biodiversity monitoring. However, despite their importance, genetic data remain underutilised in policy due to difficulties in translating technical results for decision‐makers (Jenkins and Stevens 2018; Sandström et al. 2019; Shafer et al. 2015). To help bridge this ‘conservation genetics gap’ (Taylor et al. 2017), our project was developed in partnership with Natural England and Natural Resources Wales, both of whom advise on the natural environment and have a central role in shaping policies to protect species and habitats of conservation priority within their respective nations.

Coralline algae are calcareous red seaweeds that occur in most coastal ecosystems worldwide and may function as important ecosystem engineers (Schubert et al. 2020). Free‐living, unattached sporophytes of coralline algae can accumulate into extensive three‐dimensional seabed habitats, known as maerl or rhodolith beds, that support diverse communities (Kamenos et al. 2004; Peña et al. 2014; Tuya et al. 2023) and are important blue carbon ecosystems (James et al. 2024; Mao et al. 2020). Maerl, however, grows extremely slowly (0.5–1.5 mm per thallus tip per year; Blake and Maggs 2003), and is vulnerable to disturbance, sedimentation, pollution and climate change (Bernard et al. 2019; Hall‐Spencer 2000; Legrand et al. 2024; Qui‐Minet et al. 2019). Moreover, despite their ecological importance, maerl beds remain relatively understudied compared with other marine habitats (Tuya et al. 2023).

Previous genetic studies on maerl have largely focused on DNA barcoding, which has been crucial for resolving species boundaries given the difficulty of morphological identification (Pardo et al. 2014). These data have revealed that *Phymatolithon calcareum* and *Lithothamnion corallioides* are the dominant maerl‐forming species in the NE Atlantic (Carro et al. 2014; Melbourne et al. 2017; Pardo et al. 2014, 2017). More recent work using microsatellites and whole genome genotyping provided first insights into clonality and genetic differentiation in *P. calcareum* (Jenkins et al. 2021; Pardo et al. 2019), but sampling within Britain has until now been limited, and *L. corallioides* has yet to be investigated with genomic methods.

The reproductive biology of maerl‐forming species complicates the interpretation of population genetic patterns. *P. calcareum* has a haplodiplontic life cycle with both attached gametophytes and unattached sporophytes (Pardo et al. 2019). The life cycle of *L. corallioides* has not yet been directly studied, but it is thought to resemble that of *P. calcareum* . Partial clonality means sporophytes can fragment and regrow, producing genetically identical individuals (ramets) that together form clonal lineages (genets). At the same time, sexual recruitment may contribute new genotypes, but its relative importance compared to clonal reproduction remains unresolved in *P. calcareum* and other maerl‐forming species. Indeed, the absence of gametophytes from sites sampled across the north‐west Atlantic, combined with the low occurrence of spore‐producing samples, suggests that *P. calcareum* primarily propagates through clonal fragmentation (Pardo et al. 2019). Somatic mutations add another layer of complexity, as they can generate genetic variation within large clonal lineages and potentially contribute to adaptation (Nimbs et al. 2024; Reusch et al. 2021; Stoeckel et al. 2021). One advantage of adopting genomic approaches is the ability to detect somatic mutations, enabling more accurate identification of ramets and genets, as well as clonal and genetic diversity within populations.

In England and Wales, maerl bed habitats and the two main species, *P. calcareum* and *L. corallioides* , are protected under the NERC Act 2006 and the Conservation of Habitats and Species Regulations 2017. Several marine protected areas now include maerl beds as designated features, but little is known about their genetic composition and resilience. In England, recent surveys by Natural England have provided new information on the locations of maerl beds in southern and south‐west England (Envision Marine LTD 2024), for which genomic profiling would circumvent the sampling limitations apparent in previous genetic studies of maerl across this region. In this study, therefore, we assembled draft reference genomes for both species and combined new genomic data from England and Wales with existing data (Jenkins et al. 2021) to investigate the following research questions: (i) What are the levels of clonal diversity, genetic diversity and population structure across sites and species? (ii) Is there evidence of adaptation to environmental gradients? (iii) What is the potential risk of maladaptation under future climate change scenarios?

## 2 Materials and Methods

### 2.1 Sampling Sites and DNA Extraction

**FIGURE 1:**
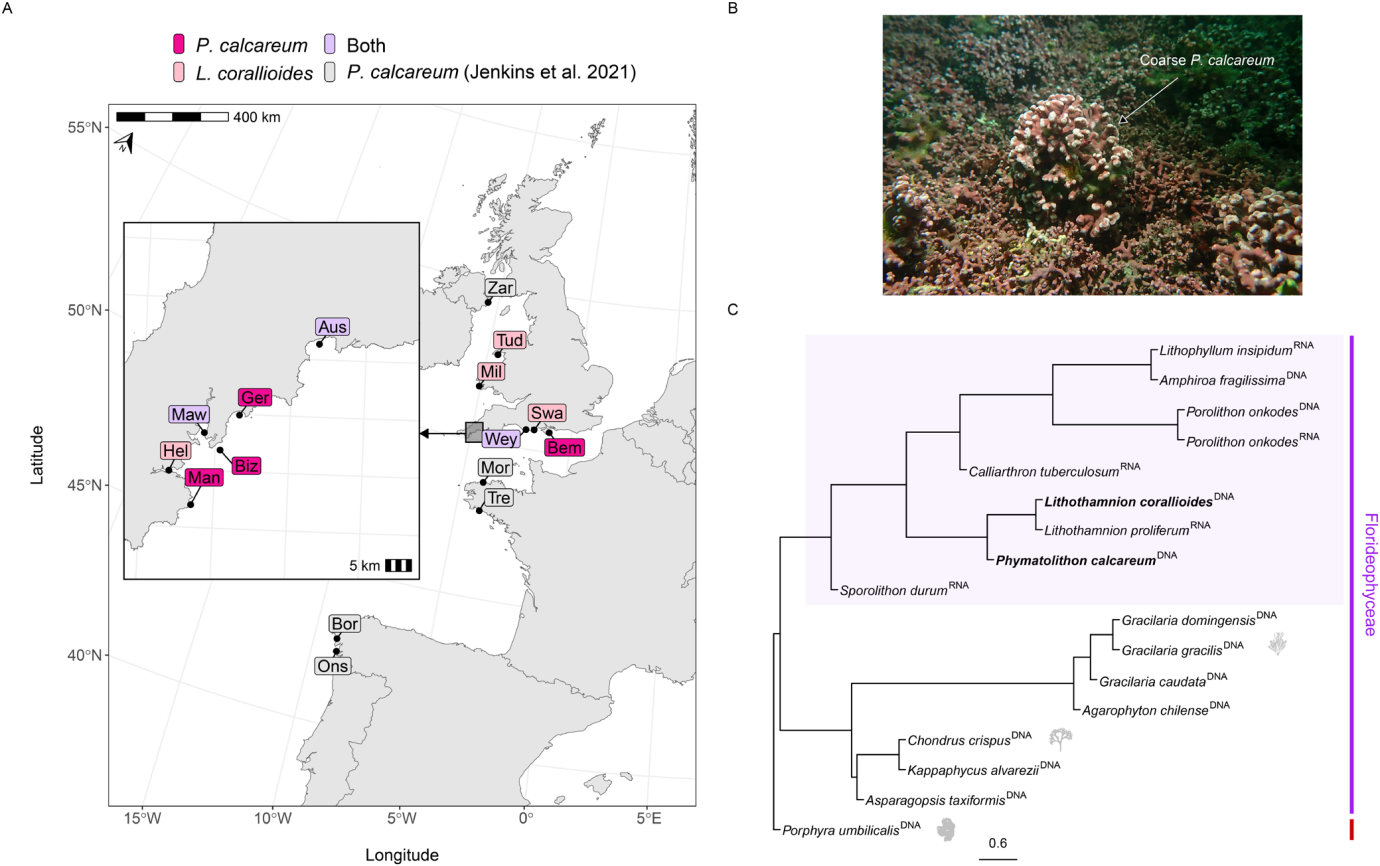
(A) Map of sites where maerl was sampled. Colours indicate the maerl‐forming species identified at each site. (B) Photo of the St Mawes maerl bed (credit: Matt Slater, Cornwall Wildlife Trust). The white arrow highlights the coarse growth form of *Phymatolithon calcareum* (MawC); surrounding maerl consists of non‐coarse *P. calcareum* and *Lithothamnion corallioides*. (C) Species tree of Florideophyceae (purple) with Bangiophyceae as an outgroup (red), built from 157 BUSCO single‐copy genes present in ≥ 10 species. The two focal species are in bold. Superscripts indicate genome (DNA) or transcriptome (RNA) assemblies. The light purple box marks calcifying Corallinophycidae lineages, including Corallinales and Hapalidiales.

Samples of maerl were collected via SCUBA diving at depths of 4–23 m from nine sites along the south‐west coast of England, and from three sites in south‐west Wales and north Wales; of these, only two had been previously assessed for maerl species identification or genetic analysis (Table 1, Figure 1). Collection of samples in both non‐protected and protected marine sites was coordinated in partnership with Natural England and Natural Resources Wales to ensure all sampling complied with priority species and habitat regulations. Transects were deployed and samples were collected at least 1 m apart to avoid potential over‐sampling of locally dominant clonal lineages, with the aim of maximising the species and genetic variation captured from each maerl bed. At sites where live maerl was more scattered and patchily distributed, samples were collected opportunistically at least 1 m apart during dives. The number of samples collected per site varied depending on live maerl bed density. All maerl samples were fully immersed in 95%–100% ethanol as soon as possible after surfacing, and ethanol was replaced after 24 h. Samples were stored at 4°C until DNA extraction.

**TABLE 1:** Summary of maerl sampling sites. The number of genets (multilocus lineages) and ramets (multilocus genotypes) is reported for *Phymatolithon calcareum* and *Lithothamnion corallioides*. For each species, clonality diversity and genetic diversity statistics for ramets are shown.

Sampling site	Year	Depth (m)	Code	Genets (MLLs)	Ramets (MLGs)	Clonal diversity		Ramets* genetic diversity		
						λ	Pareto β	*H*_O_	*H*_S_	*F*_IS_
*Phymatolithon calcareum*										
Cornwall, St Austell Bay	2022–2023	5–18	Aus	7	10	0.73	1.78	0.28	0.23	−0.22
Cornwall, Falmouth, The Bizzies	2022	23	Biz	8	10	0.77	2.00	0.26	0.21	−0.22
Cornwall, Falmouth, St Mawes	2015, 2022 (REF)	4–6	Maw	6	9	0.71	1.63	0.31	0.22	−0.35
Cornwall, Falmouth, St Mawes ^COARSE^	2011, 2022	4–6	MawC	3	15	0.22	0.43	0.65	0.34	−0.85
Cornwall, Gerrans Bay	2022–2023	13–18	Ger	6	15	0.56	0.86	0.28	0.20	−0.33
Cornwall, The Manacles	2015	5–10	Man	10	12	0.76	2.10	0.26	0.21	−0.20
Dorset, Weymouth	2022	15–20	Wey	3	5	0.56	1.00	0.28	0.19	−0.39
Isle of Wight, Bembridge	2022	13–21	Bem^+^	—	—	—	—	—	—	—
*Lithothamnion corallioides*										
Cornwall, St Austell Bay	2022–2023	5–18	Aus	14	14	0.80	*Inf*	0.34	0.29	−0.11
Cornwall, Falmouth, St Mawes	2015, 2022 (REF)	4–6	Maw	4	5	0.72	2.00	0.37	0.30	−0.25
Cornwall, Helford Estuary	2015	5–10	Hel	6	13	0.56	0.88	0.39	0.29	−0.25
Dorset, Swanage	2022	15–19	Swa^+^	3	3	—	—	—	—	—
Dorset, Weymouth	2022	15–20	Wey^+^	4	4	—	—	—	—	—
Pembrokeshire, Milford Haven west	2023	5	Mil1	11	13	0.77	2.18	0.38	0.30	−0.19
Pembrokeshire, Milford Haven east	2023	5	Mil2	11	12	0.79	3.46	0.39	0.30	−0.21
Llyn Peninsula, St Tudwal's Island	2023	12	Tud^+^	2	2	—	—	—	—	—

*Note:* (REF) reference genome was assembled for one individual sample using Nanopore long‐read sequencing. MLL, multilocus lineage (proxy for the number of genets); MLG, multilocus genotypes (proxy for the number of ramets); λ, Simpson's diversity index; *Inf*, infinity denotes when Pareto β cannot be computed because no clones are present (the number of genets equals the number of ramets).

^+^ In Bembridge, sequencing data identified five out of 10 samples to species level. The quality of data was insufficient for population genomics analysis, likely due to very low coverage of live algae on samples. Other sites had insufficient sample size (*N* < 5) to compute site‐level diversity statistics.

* See Table S4 for genetic diversity of both genets and ramets.

Genomic DNA was extracted using the DNeasy Blood and Tissue Kit (Qiagen), with some modifications to the protocol designed to increase DNA yield from maerl samples (see Figure S1 for detailed protocol). This was required, particularly for Nanopore sequencing, because the outer layer of live algae is very thin and typically yields extremely low amounts of DNA using the standard protocol. The quantity and concentration of DNA were assessed by fluorometry using a Qubit 1X dsDNA High Sensitivity Assay Kit (Invitrogen). The quality of each aliquot was checked using gel electrophoresis on a 1% agarose gel, and only DNA samples showing a ~20 Kb band or higher with low or no evidence of degradation were selected for whole‐genome sequencing.

### 2.2 Whole‐Genome Sequencing

Illumina short‐read libraries were prepared for all samples using NEBNext Ultra II FS following the protocol for inputs below 50 ng (half reactions were performed). Adaptors were diluted 1:10 for all samples as per the protocol. No size selection was implemented, and 0.8X bead purification was conducted twice prior to seven cycles of PCR enrichment. Libraries were checked by TapeStation D1000 and pooled equimolar. All samples were sequenced on a NovaSeq 6000 using a paired‐end strategy with a read length of 150 bases. Additionally, WGS data for maerl samples from a previous study (Jenkins et al. 2021) were included in our study (Figure 1); these DNA samples were prepared and sequenced using the same methods described above. Illumina raw reads were filtered and trimmed using fastp v0.23.4 (Chen 2023) with the following parameters: (i) polyG tail trimming enabled, (ii) a Phred quality score > = 30 in at least 60% of bases in a read and (iii) a minimum length of 100 bp.

Nanopore long‐read libraries were prepared for two samples, one representing *P. calcareum* and one representing *L. corallioides* , both of which were confirmed to be the correct species by DNA barcoding (see below) and originated from St Mawes, Falmouth (Table 1). For both samples, the same DNA aliquot used to prepare the Illumina libraries was used to prepare Nanopore libraries so that the Illumina data could be used to polish the genome assembled with long reads. Standard Oxford Nanopore Technology libraries were prepared and sequenced on a PromethION 24 flow cell with R9.4.1 chemistry. Guppy v6.3.9 with super‐accurate basecalling was used for basecalling, and only reads flagged as passed (minimum quality score of 10) were retained in FASTQ format. Porechop v0.2.4 was used to detect and remove adapters from reads passing the basecalling filters.

### 2.3 Species Identification via DNA Barcoding

A DNA barcoding approach was used to confirm species identity of each sample; this is necessary because of the difficulty in distinguishing maerl‐forming species based on external morphology (Pardo et al. 2014). First, published mitochondrial and chloroplast genomes of *P. calcareum* (MW357900.2, OQ417768.1) and *L. corallioides* (MW357901.2, OQ417769.1) were downloaded from GenBank in FASTA format to function as seed sequences for each organelle. The GenBank reference files for each of these accessions were also downloaded for use in the organelle gene annotation step. Second, the Illumina trimmed reads for each sample were aligned to the mitogenome and plastome seed sequences separately using Bowtie2 v2.5.3 (Langmead and Salzberg 2012). Mapped reads were extracted from each BAM file to a FASTQ file using SAMtools v1.19 (Danecek et al. 2021). These target reads were then assembled using Unicycler v0.5.0 (Wick et al. 2017), with the aim of obtaining a complete mitochondrial and chloroplast genome for each individual sample. Third, for organelle genomes successfully assembled, both mitochondrial and chloroplast genomes were annotated using a custom Python script (see data archiving statement) using the GenBank reference files. Lastly, for each sample, nucleotide coding sequences (CDS) of the *COI* mitochondrial gene and the *psbA* chloroplast gene were extracted to generate two FASTA files containing CDS for *COI* and *psbA*, respectively. These genes were chosen because they are the most common genes used in coralline algae DNA barcoding studies (Pardo et al. 2014, 2017; Peña et al. 2020), and have the highest intra‐ and interspecies representation on GenBank. These two FASTA files were submitted to the NCBI *blastn* online server to compare our CDS (query) to the nucleotide database (subject). For a given maerl sample, a species identification was assigned if the percentage identity was ≥ 99% (Carro et al. 2014) and the localities of *P. calcareum* and *L. corallioides* query accessions are within the geographic range of our samples: the United Kingdom, Ireland, France and Spain.

### 2.4 Genome Assembly, QC and Contig Filtering

A reference genome assembly was built for both *P. calcareum* and *L. corallioides* using Nanopore long reads. Prior to assembly, long reads were taxonomically classified using BugSeq (Fan et al. 2021), which classifies reads against the BugSeq default microbial database. Because of the potential for non‐target DNA to be isolated from the sample during extraction, that is, microbes living on or inside the calcium carbonate skeletal structure of maerl, the rationale here was to identify and remove as many reads of microbial origin as possible prior to the assembly pipeline. From the classification results, only reads classified as 0 (unclassified) or 1 (other) were retained, thus removing all reads classified as Bacteria, Virus or other microbes in the database. Flye v2.9.3 (Kolmogorov et al. 2019) was used to assemble the reads into contigs. The input read length was adjusted to explore the optimum minimum read length for input into Flye; for both samples ≥ 3000 bp was deemed the optimum. For Flye, default parameters were used, alternative contigs were not kept (−no‐alt‐contigs), and only contigs longer than 3000 bp were retained. The assemblies were polished with Illumina short read data using Polypolish v0.6.0 (Wick and Holt 2022).

BlobToolKit v4.2.1 (Challis et al. 2020) was used to assess contamination and quality of the assemblies. Both *blastn* v2.14.1+ (Camacho et al. 2009) and *diamond blastx* v2.1.8 (Buchfink et al. 2021) were run using the assemblies as queries to the NCBI nucleotide and protein databases, respectively. In addition, minimap2 v2.26 (Li 2018) was used to map Nanopore reads to each assembly. The taxonomic hits and sorted BAM alignments, along with the assembly FASTA file, were used as inputs to the *blobtools create* command to create a BlobDir. Contigs were removed from the assembly if the following conditions were met: (i) contig was of organelle genome origin, (ii) contig was not supported by mapped reads (at least one read supporting each base across the contig) and (iii) contig matched to Bacteria, Archaea, Virus, or any Eukaryote other than Rhodophyta or Streptophyta. Assembly statistics, including total length, number of contigs, N50 and GC content, were computed using the *stats* command from *seqkit* v2.5.1 (Shen et al. 2016).

### 2.5 BUSCO Phylogeny

BUSCO v5.6.1 (Manni et al. 2021) was executed using the eukaryota_odb10 dataset to assess the completeness of near‐universal single‐copy orthologs in both genome assemblies. Subsequently, a phylogeny of coralline algae (and several other red algal taxa) was built using BUSCO protein sequences, the rationale of which was twofold: (i) to examine the tree position of *P. calcareum* and *L. corallioides* to further confirm the validity of the genome assemblies, and (ii) to compute and visualise a phylogeny of coralline algae based on BUSCO genes. Genome and/or transcriptome assemblies were downloaded for all available coralline algae and several red algal species from the Florideophyceae (Table S1). A BUSCO analysis was run for each assembly, and the outputs were supplied as inputs to a modified version of the BUSCO phylogenomics pipeline (see data archiving statement). This pipeline identifies complete single‐copy BUSCOs present in at least 10 assemblies, then aligns sequences, trims alignments and constructs gene trees using IQ‐TREE (Nguyen et al. 2015). Finally, Astral v5.7.8 (Mirarab and Warnow 2015) was used to build a coalescent‐based species tree, with *Porphyra umbilicalis* (Bangiophyceae) used as the outgroup for visualisation.

### 2.6 Variant Calling and SNP Filtering

After each DNA sample was identified to species level, the trimmed Illumina reads were mapped to the *P. calcareum* or *L. corallioides* genome assemblies using Bowtie2 v2.5.3 (Langmead and Salzberg 2012). The alignments were sorted by coordinate, then read groups were added and duplicates were marked using GATK v4.5 (McKenna et al. 2010). Variant calling was conducted with Freebayes v1.3.8 (Garrison and Marth 2012) in parallel on 100,000 bp chunks using *freebayes‐parallel* and the *fasta_generate_regions.py* script. The following parameters were used: minimum mapping quality > = 20, minimum base quality > = 30, compute genotype qualities, and the ploidy was set to two. BCFtools v1.21 (Danecek et al. 2021) and VCFtools v0.1.16 (Danecek et al. 2011) were used to filter variants. First, poor quality variants were removed by enforcing a maximum missing threshold of 0.70, a minor allele count of 3 and a minimum quality score of 30. Second, all samples from Bembridge were removed due to a very high proportion (50%–95%) of missing genotypes, likely because of insufficient DNA extracted from the target species (Bembridge samples comprised mostly dead maerl). Third, the following parameters were enforced using BCFtools: (i) no missing data, (ii) genotype quality > = 20, (iii) minimum depth > =3, (iv) allele depth > = 10, minor allele frequency > = 5% and allele balance > = 0.10. Lastly, the VCF was read into R v4.3.2 and the following filters were applied to create a high quality biallelic SNP dataset: (i) distance thinning such that SNPs within 1000 bp on a given contig were removed (to avoid potential linkage), (ii) SNPs with a minimum depth of 15 and a maximum depth of 100 over all samples were removed and (iii) monomorphic loci were removed.

### 2.7 Ploidy

Ploidy of each sample was assessed using the *determining ploidy 1* method outlined in the vcfR R package documentation (Knaus and Grünwald 2017). This method examines allele balance information, that is, the number of times an allele was sequenced, at each heterozygous SNP. In diploids, an allele frequency ratio of 50:50 is expected (both alleles are sequenced equally), although there is inevitably variation around this ratio introduced by sequencing bias and variant calling software. When the allele frequencies of all heterozygous SNPs are visualised, we would expect a peak at 1/2. In contrast, in triploids, we would expect two peaks at 1/3 and 2/3. Preliminary analysis of allele balance indicated that read depth and minimum allele depth were important for interpreting the histograms; so, prior to analysis, SNPs were removed where the depth of at least one allele was < 10, and only samples with a median read depth ≥ 30 were considered for ploidy determination.

### 2.8 Clonality and Genetic Diversity

Clonality and genetic diversity were investigated for each species for each maerl bed separately. Clonal lineages, defined by multilocus lineages (MLLs) and used as a proxy for the number of genets, and the number of clones, defined by multilocus genotypes (MLGs) and used as a proxy for the number of ramets, were assessed using the R package poppr v2.9.6 (Kamvar et al. 2014). First, pairwise Hamming genetic distances were calculated between all samples. Second, *cutoff_predictor* *()* was run to define a clone threshold based on the genetic distance matrix. Defining a clone threshold is necessary because somatic mutations can introduce small genetic differences between clones. By implementing stringent SNP filtering criteria based on no missing data and depth, we increased the likelihood that identified clones are genuine and not the result of sequencing errors. Lastly, *mlg.filter()* was run using the distance matrix, clone cut‐off threshold and the average neighbour algorithm to identify MLLs (genets). To assess clonal diversity, the Simpson's index (λ) and the negative slope of the Pareto distribution (β) were computed using the *diversity_ci* *()* function from poppr using the rarefy parameter to control for different sample sizes. Simpson's index (λ) ranges from 0 to 1 and represents the probability that two individuals drawn at random belong to different clonal lineages (Meirmans 2025). Pareto β characterises clonal diversity and skewness in the distribution of clones within a population; the presence of a few large clonal lineages and many small ones will result in lower β values, while more balanced lineages of similar sizes will result in higher β values (Arnaud‐Haond et al. 2007). Observed heterozygosity (*H*_o_), expected heterozygosity (*H*_s_) and *F*_IS_ (inbreeding coefficient) were computed for both genets (MLLs only) and ramets (all sequenced samples, MLGs).

### 2.9 Population Structure

Clone‐correction has often been applied in population genetic analyses of partially clonal organisms, where only a single representative of an MLL is retained to prevent bias thought to be apparent (Meirmans 2025). However, while differences between *H*_s_ and *F*_IS_ can be informative about the rates of sexual versus asexual reproduction, clone‐correction has been demonstrated to severely underestimate genetic differentiation (*F*_ST_) and, thus, interpretations of population structure (Meirmans 2025). Moreover, genetic variation can arise during clonal reproduction through somatic mutations and be potentially adaptive (Nimbs et al. 2024; Reusch et al. 2021; Yu et al. 2020). All subsequent analyses were, therefore, performed using multi‐locus genotypes (MLGs), as a proxy for the ramets present at a site.

Population structure was assessed using non‐model–based principal component analysis (PCA) and model‐based admixture inference using PopCluster v1.4.0 (Wang 2022). For admixture analyses, the admixture model with unequal allele frequencies and medium scaling was run independently 10 times for each ancestral population (*K*). No sample grouping or site of origin information (priors) was supplied to the algorithm. The optimal run and *K* statistics (determined by *D*_*LK*2_ and *F*_*STIS*_ statistics) (Wang 2022) were extracted from the results. Individual admixture proportions were visualised as barplots, and the mean average proportions per cluster per site were visualised as admixture maps using mapmixture v1.2.0 (Jenkins 2024). Sites that had fewer than three samples were not visualised in the admixture maps. Genetic differentiation between sites was assessed by computing pairwise *F*_ST_ values (Weir and Cockerham 1984).

### 2.10 Genotype–Environment Association (GEA)

Environmental data were downloaded from Bio‐Oracle v3.0 (Assis et al. 2024) at a resolution of 0.05 degrees (~5.5 km at the equator). NetCDF files were obtained for environmental variables which have been shown or inferred to be important for maerl growth and survival (Martin and Hall‐Spencer 2017): Ocean temperature (°C), salinity (PSU), pH, oxygen concentration (mmol m^−3^) and seawater velocity (m s^−1^). For each environmental variable, the following parameters were selected: Baseline (2010–2020), benthic (mean average depth per cell) and variable mean (mean average across the time period). Future layers (2050–2060) for these variables based on the Shared Socioeconomic Pathway (SSP) SSP245 (middle of the road) scenario from CMIP6 were also obtained. For each sample, latitude and longitude coordinates were used to extract values for each variable to use as a set of predictor variables in the analysis. There were only very small differences in pH among sites (8.06–8.08), while multicollinearity checks among the variables revealed a very high correlation between oxygen concentration and ocean temperature (*r* ^2^ = −0.96). Therefore, only temperature, salinity and seawater velocity were retained for genotype‐environment association (GEA) analyses. To mitigate spatial autocorrelation, detrending of environmental variables was conducted prior to modelling. First, least‐cost geographic distances along coasts were calculated between sites, and the output was used to compute dbMEMs (distance‐based Moran's eigenvector maps). Second, a linear model for each environmental variable was modelled as a function of the dbMEMs and the residuals were extracted. The residuals, representing the detrended environmental variables, were used as predictors in the GEA model.

A partial redundancy analysis (RDA) was performed using the R package vegan v2.6–10 with the SNP genotypes as the dependent variable. To control for population structure and isolation‐by‐distance, the first two principal components of a PCA on the genotypes and the dbMEMs described above were used as conditions in the model, respectively. Outlier SNPs, that is, SNPs whose genotypes correlate significantly with the environmental predictors, were identified based on their extremeness along a distribution of Mahalanobis distances estimated between each locus and the centre of the RDA space using three axes (Capblancq et al. 2018; Capblancq and Forester 2021). The Bonferroni correction was used to account for multiple testing, and SNPs with adjusted *p* values < 0.01 were considered as outlier SNPs. For *L. corallioides* , only one outlier SNP was detected, so no further GEA or offset analyses were conducted with *L. corallioides* .

### 2.11 Genomic Offset

Outlier SNP genotypes were used as input to the *genetic.offset* *()* function from the LEA R package. A matrix of climate data representing both present‐day baseline environments (2010–2020) and future environments (2050–5060) was used as covariates. This function calculates a geometric genomic offset value for each site using a genetic gap algorithm (Gain et al. 2023). In a population genetic context, the geometric genomic offset can be interpreted as the average value of Nei's *D*_ST_ (divided by two) for the set of loci assumed to be involved in local adaptation (Gain et al. 2023). Offset estimates predict the relative allele frequency change needed for a population to lower its risk of being potentially maladapted to future environments.

## 3 Results

### 3.1 Species Identification

DNA barcoding analysis using *COX1* and *psbA* identified 86 as *Phymatolithon calcareum* and 66 as *Lithothamnion corallioides* (Table 1). Five samples from Bembridge could not be identified to species. Elsewhere in Britain, there were some sites where all samples were identified to be a single species, while at other sites, samples of both species were found (Figure 1A).

### 3.2 Draft Reference Genome and BUSCO Phylogeny

A draft reference genome was assembled for each species using Nanopore long reads. The assembly statistics were comparable to or improved upon the two coralline red algal species, *Amphiroa fragilissima* and *Porolithon onkodes*, for which draft reference genomes are currently available (Table S2). For *P. calcareum* , the reference genome was 150 Mbp in length, 6375 contigs, with an N50 of 35 Kbp and an overall GC content of 43%. For *L. corallioides* , the reference genome was 145 Mbp in length, 9939 contigs, with an N50 of 19 Kbp and an overall GC content of 40%. Using the Eukaryota orthoDB v10 database (*N* = 255), the BUSCO results for *P. calcareum* were C:62.7% [S:59.6%, D:3.1%], F:12.5%, M:24.8%; and for *L. corallioides* were C: 40.8% [S:39.2%, D:1.6%], F:11.8%, M:47.4%. The species tree of coralline algae and other Florideophyceae red algal species was built using 157 concatenated BUSCO genes that were complete and single‐copy in at least 10 species (Figure 1C). This tree showed that all coralline algae were positioned on a branch separate from all other Florideophyceae species. Within this branch, the evolutionary relationships amongst species were as expected based on previous research on coralline algae phylogenetics (Peña et al. 2020). For the maerl‐forming species in our study, the tree (Figure 1C) showed that both *Lithothamnion* species, *L. corallioides* and *L. proliferum*, were monophyletic, sharing a recent common ancestor, and that both these species shared their most recent common ancestor with *P. calcareum* .

### 3.3 SNP Dataset Summary

For *P. calcareum* , filtering the raw variant calls from Freebayes resulted in 124,751 SNPs. Five samples were removed at this stage because of very high missing data (> 95%); these samples were all from Bembridge, which is probably because these samples had very little live algal coverage. Further filtering of these variants by distance thinning (linkage disequilibrium), mean read depth and keeping only biallelic SNPs resulted in a final dataset of 76 samples genotyped at 15,330 SNPs for *P. calcareum* (130 samples including sites from Jenkins et al. 2021). For *L. corallioides* , filtering the raw variant calls from Freebayes resulted in 192,041 SNPs. Further filtering of these variants resulted in a final dataset of 66 samples genotyped at 10,215 SNPs for *L. corallioides* .

### 3.4 Triploidy in *P. calcareum* Coarse Growth Form

**FIGURE 2:**
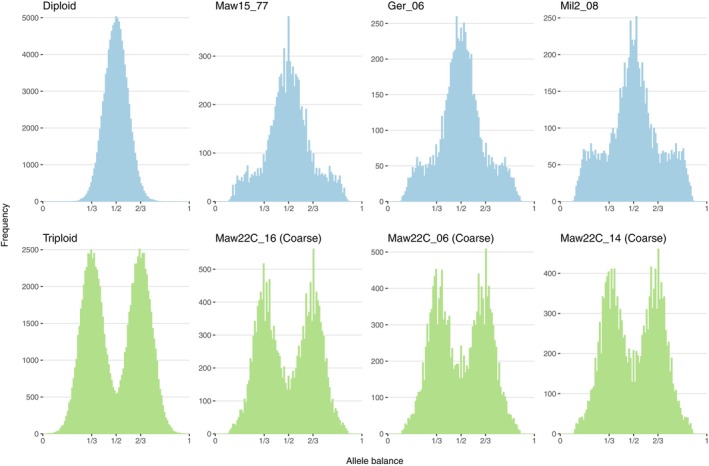
Ploidy analysis based on allele balance proportions. The first column shows allele frequency histograms of the expected distribution for a diploid (single peak at 1/2) and a triploid (two peaks at 1/3 and 2/3). The remaining columns show histograms of allele balance proportions from heterozygous SNPs in samples from St Mawes ( *Phymatolithon calcareum* ), Gerrans Bay ( *P. calcareum* ) and Milford Haven (*Lithothamnion corallioides*); coarse *P. calcareum* maerl samples are presented on the bottom panel. Colours denote whether the pattern is indicative of diploidy (blue) or triploidy (green).

At the St Mawes maerl bed, we found two growth forms, both identified as *P. calcareum* by DNA barcoding, one of which was typically around 2 cm in diameter with characteristic delicate thalli, while the other was typified by a much larger rhodolith (4–5 cm in diameter) with far bulkier branching structures (Figures 1 and S2). Hereafter, we refer to the latter growth form in St Mawes as the ‘coarse’ form (MawC). Allele balance analysis of all coarse maerl samples showed a clear pattern of triploidy (Figure 2), with two peaks at 1/3 and 2/3 as per expectations of triploids. These samples were compared with all other *P. calcareum* samples which demonstrated that all other samples were diploid, as evidenced by a single peak for allele balance at 1/2 (Figure 2). All *L. corallioides* samples with sufficient median read depth showed a peak at 1/2 and were deemed to be diploids.

### 3.5 Clonal Lineages and Genetic Diversity

**FIGURE 3:**
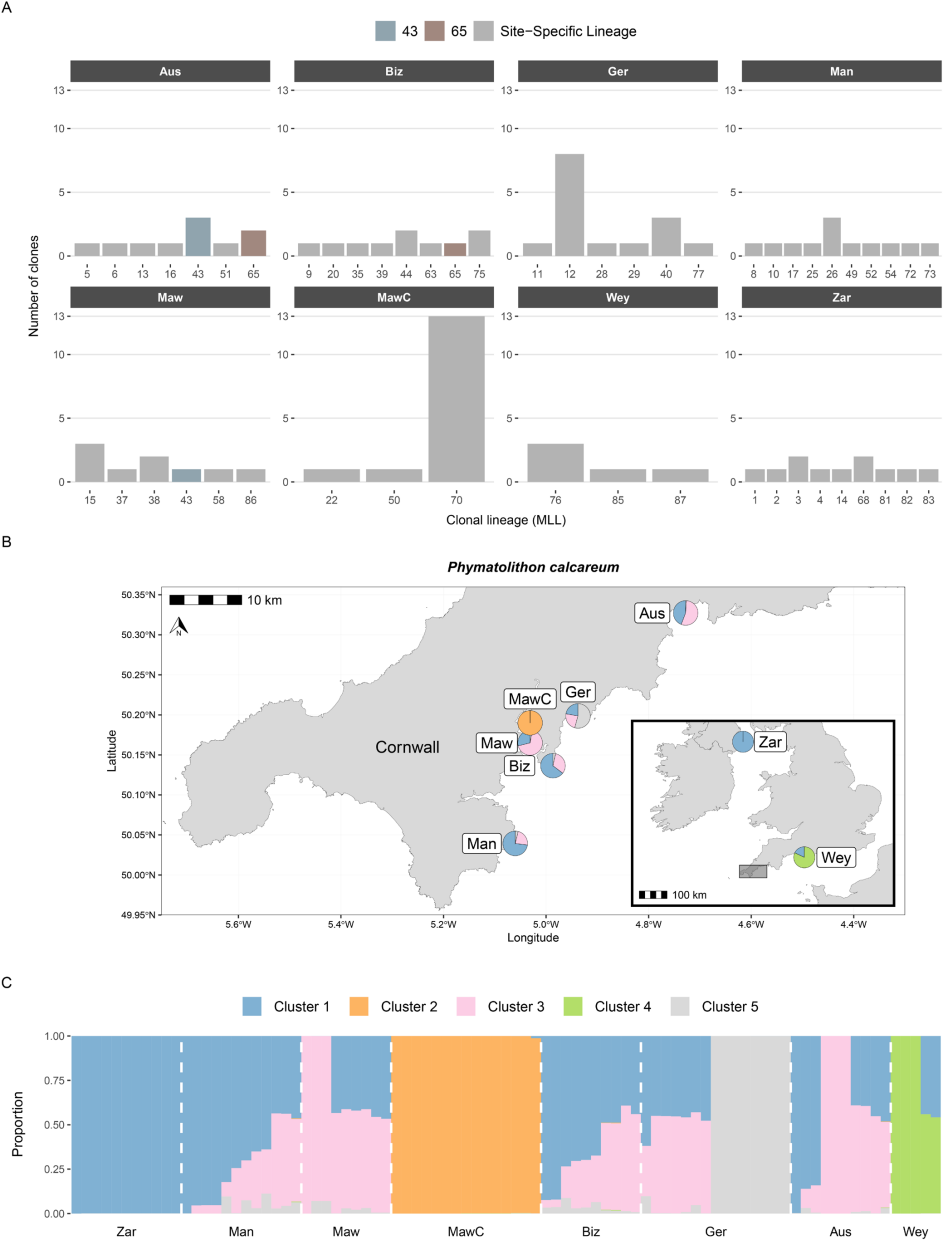
(A) *Phymatolithon calcareum* multilocus lineages (MLL), a proxy for the number of genets, and the number of clones, a proxy for the number of ramets. Grey bars indicate that a clonal lineage is only present in that site, whereas coloured bars indicate that a lineage was found in multiple sites. (B) *P. calcareum* admixture map, where each pie represents the average admixture proportion across all samples for a given cluster at a given site. (C) *P. calcareum* structure plot, where each bar represents the proportion of a sample's genome derived from each *K* source ancestral population (genetic cluster).

**FIGURE 4:**
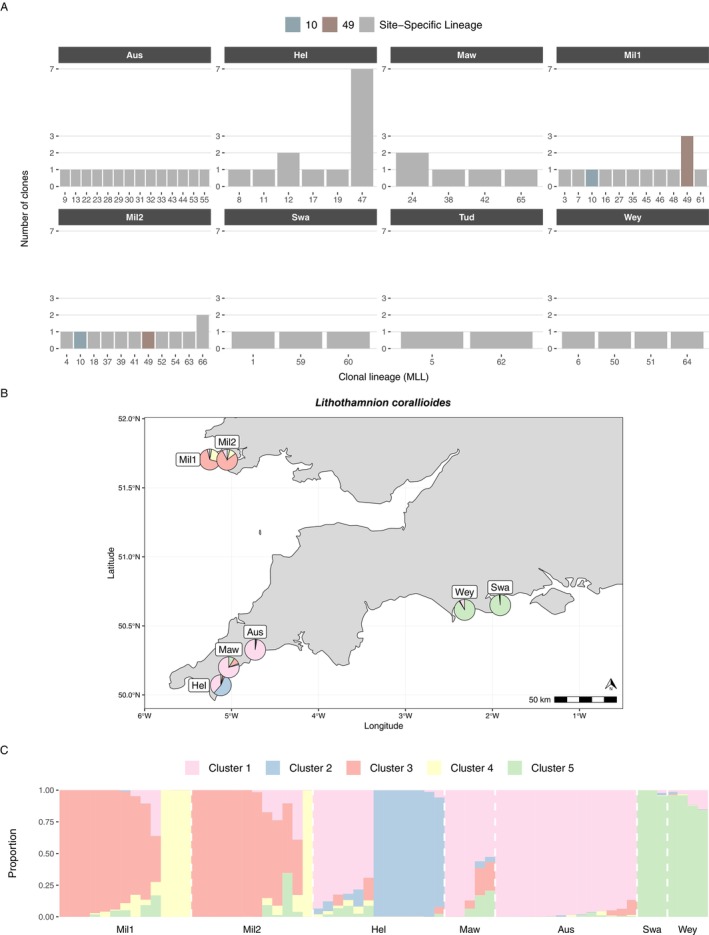
(A) *Lithothamnion corallioides* multi‐locus lineages (MLL), a proxy for the number of genets, and the number of clones, a proxy for the number of ramets. Grey bars indicate that a clonal lineage is only present in that site, whereas coloured bars indicate that a lineage was found in multiple sites. (B) *L. corallioides* admixture map, where each pie represents the average admixture proportion across all samples for a given cluster at a given site. (C) *L. corallioides* structure plot, where each bar represents the proportion of a sample's genome derived from each *K* source ancestral population (genetic cluster).

Clonality and genetic diversity are reported for *P. calcareum* from sites in Britain (*N* = 87), as data from sites in France and Spain have been analysed in a previous study (Jenkins et al. 2021). Based on Hamming genetic distances, the clone cut‐off threshold (i.e., the threshold used to define samples as clones of each other), was estimated as 0.054 in *P. calcareum* and 0.125 in *L. corallioides* (Figure S3). For *P. calcareum* , this translated to 50 clonal lineages/genets (out of 87 samples, including Zara Shoal, Northern Ireland) (Figure 3A). For *L. corallioides* , this translated to 53 clonal lineages/genets (out of 66 samples) (Figure 4A).

For *P. calcareum* , clonality diversity was highest in The Manacles and The Bizzies and lowest in the coarse growth form in St Mawes (MawC), the latter of which also showed the highest levels of observed and expected heterozygosity (Table 1). A large clonal lineage was identified in coarse maerl at St Mawes (MLL70) and in Gerrans Bay (MLL12). Two clonal lineages were found across two different sites: MLL43 was found in both St Austell Bay and at St Mawes, and MLG65 was found in both St Austell Bay and at The Bizzies. Observed heterozygosity in *P calcareum* ramets ranged from 0.26 to 0.65, while expected heterozygosity ranged from 0.19 to 0.34, and all values of *F*_IS_ were negative. Genetic diversity values for *P. calcareum* genets were very similar to the values for ramets (Figure S4).

For *L. corallioides* , clonal diversity was high at all sites except the Helford Estuary, where one large clonal lineage (MLL47) and five smaller lineages were identified. In St Austell Bay, no clones were detected, meaning every sample represented a potentially distinct clonal lineage. Observed heterozygosity in *L. corallioides* ranged from 0.34 to 0.39, while expected heterozygosity ranged from 0.29 to 0.30, and all values of *F*_IS_ were negative. Genetic diversity values for *L. corallioides* genets were also very similar to the values for ramets (Figure S4).

### 3.6 Population Structure

For *P. calcareum* , PopCluster statistics (*D*_*LK*2_ and *F*_*STIS*_) showed the highest support for five *K* ancestral populations (genetic clusters) (Figures S5, S6), so *K* = 5 was used for visualising the admixture map and structure plot (Figure 3B,C). Three of the five clusters were largely confined to single sites: the orange cluster to coarse maerl in St Mawes, the grey cluster to Gerrans Bay (all samples from MLL12), and the green cluster to Weymouth. The remaining blue and pink clusters were admixed across The Manacles, St Mawes (noncoarse maerl), The Bizzies, Gerrans Bay and St Austell Bay (all sites along the south coast of Cornwall), while Zara Shoal in Northern Ireland showed shared ancestry with these sites (blue cluster) but no admixture. These patterns of population structure were supported by PCA when visualising the first three principal components (Figure S7).

For *L. corallioides* , PopCluster analyses showed a similar range of structuring at different values of *K*, with *K* = 5 and *K* = 6 showing the highest statistical support (Figures S8, S9). Since there was support for five genetic clusters in the PCA (Figure S10), *K* = 5 was used for visualising the admixture map and structure plot (Figure 4B,C). Four of the five clusters (Figure 4B,C) were largely restricted to specific sites or regions: The yellow cluster (all samples from MLL34) and red cluster in Milford Haven, the blue cluster in the Helford Estuary (all samples from MLL47), and the green cluster in Weymouth and Swanage. The pink cluster predominated in the Helford Estuary, St Mawes and St Austell Bay, though admixture levels varied among samples. These patterns of population structure were supported by PCA when visualising the first three principal components (Figure S10).

Pairwise genetic differentiation measures of *F*_ST_ ranged from 0.019 (Man–Biz) to 0.373 (MawC–Ger) in *P. calcareum* and from 0.001 (Mil1–Mil2) to 0.109 (Hel–Swa) in *L. corallioides* (Figures S11, S12). The most differentiated site for *P. calcareum* was the coarse triploidy maerl from St Mawes (*F*_ST_ range: 0.331–0.373), followed by Weymouth (*F*_ST_ range: 0.191–0.333), while in *L. corallioides,* the most differentiated sites were the Helford Estuary (*F*_ST_ range: 0.069–0.109) and Swanage (*F*_ST_ range: 0.022–0.0.109). Overall, patterns of pairwise *F*_ST_ emulated the patterns of population structure.

### 3.7 *Phymatolithon calcareum* GEA and Genomic Offset

**FIGURE 5:**
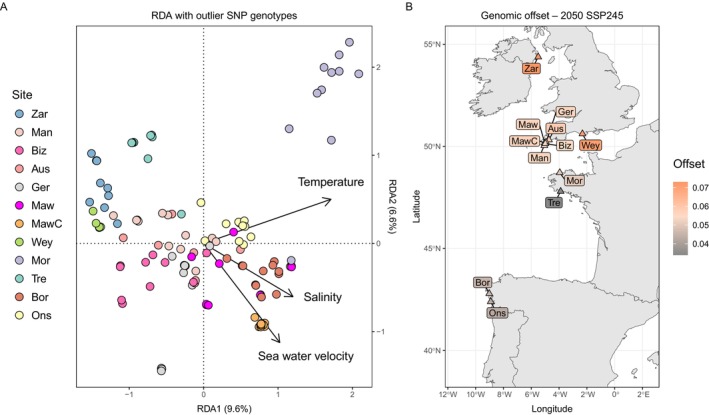
(A) *Phymatolithon calcareum* redundancy analysis (RDA) using allele frequencies of the outlier loci, and temperature, salinity and seawater velocity as the predictor variables. Each point represents a sample, and colours denote the site of origin. (B) Map of genomic offsets, averaged across samples for each site, for the mid‐century shared socioeconomic pathway (SSP) middle‐of‐the‐road climate scenario. The offset is a relative measure, meaning that the risk of being maladapted to future climates is only relevant in this analysis (not comparable across studies).

The GEA analysis included all samples, including those from France and Spain, for a total of 130 ramets genotyped at 15,330 SNPs. Using benthic ocean temperature, salinity and seawater velocity as climate predictors, 1269 outlier loci were identified as potentially climate‐associated SNPs. RDA was repeated using only the genotypes for the outlier SNPs, which explained 17.7% (adjusted *r* ^2^) of the variation in the allele frequencies (Figure 5A). This showed that samples from Zara Shoal and Weymouth were associated with lower salinities and generally lower temperatures, while samples from The Bizzies were associated with lower temperatures. In contrast, samples from Morlaix (north‐west France) and both sites in Spain (Bornalle and Illa de Ons) were associated with higher temperatures, with the Spanish sites also associated with higher salinities. Additionally, all coarse maerl samples in St Mawes (MawC) were associated with higher seawater velocities. The lowest genomic offsets for the 2050 SSP245 scenario were observed in samples from France and Spain (Trévignon, Bornalle and Illa de Ons), while the highest genomic offsets were observed in Weymouth and Zara Shoal (Figure S13). All samples in Cornwall showed very similar offsets, including within sites such as St Mawes and Gerrans Bay, where multiple genetically distinct lineages were present. Genomic offsets were averaged across samples for each site and visualised on a map (Figure 5B).

## 4 Discussion

This study provides the first draft reference genomes and a population genomics analysis of two species of coralline algae, *Phymatolithon calcareum* and *Lithothamnion corallioides*, that form maerl bed habitats in the north‐east Atlantic. Across most sites, *P. calcareum* exhibited moderate clonal diversity, with multiple genets present and relatively few repeated clones. Extreme clonality, where one genet with many repeated clones is dominant, was observed in the coarse triploid form of this species at St Mawes and in Gerrans Bay, which is indicative of asexual reproduction by fragmentation. Thus, extreme clonality appears site‐specific rather than species‐wide. In contrast, *L. corallioides* generally displayed high clonal diversity, with most samples representing unique genets. This distinction has important conservation implications. Populations dominated by a few clones may be more sensitive to environmental change because low recombination rates limit the introduction of new genetic variants. Conversely, populations with high clonal diversity or frequent sexual recruitment maintain more genetic variation, potentially enhancing adaptive capacity. The negative *F*_IS_ values detected in both species are consistent with a predominance of clonal reproduction, although occasional recombination events cannot be excluded. The prevalence and contribution of sexual reproduction could be further explored by, for example, surveying for attached gametophytes with uniporate conceptacles (Pardo et al. 2019), which remain rarely observed in maerl‐forming species (Pardo et al. 2017). Detecting such reproductive stages would have important implications, since they represent a key pathway for introducing novel genetic variants into populations, complementing other sources of variation such as somatic mutations (Reusch et al. 2021).

For *P. calcareum* , genotype–environment association analysis identified potentially climate‐associated SNPs correlated with sea temperature, salinity and seawater velocity. However, only a small proportion of genetic variation was explained, highlighting the complexity of adaptation and the influence of unmeasured environmental or biological variables. Genomic offset analyses suggested that certain sites, such as Zara Shoal and Weymouth, may require greater shifts in their allele frequencies to mitigate the risk of being maladapted to future environments under projected mid‐century climate scenarios. In comparison, sites further south in north‐west France and in north‐west Spain were predicted to have lower relative risks of maladaptation to future environments. While these insights are valuable, genomic offset predictions alone cannot fully capture the resilience of populations and species, particularly in long‐lived partially clonal organisms. In addition, predictions of maladaptation risk do not consider changes in other conditions that may have additional impacts on the habitat suitability and survival of maerl, such as increased exposure to physical disturbances, sedimentation, pollution or invasive species. Thus, determining the functional resilience of populations or individual clonal lineages will require targeted experimental approaches, such as aquaria experiments or reciprocal transplants, to evaluate which populations or lineages are more or less tolerant to climatic and environmental change.

Population structure analyses revealed strong differentiation among maerl beds, likely reflecting limited dispersal, persistence of old clonal lineages, geographic isolation and/or potential differences in colonisation history and glacial refugia (Jenkins et al. 2018, 2021; Pardo et al. 2019). For *L. corallioides* , both sites in Milford Haven were genetically differentiated from all other sites sampled in England, with some evidence of within‐site differentiation driven by the presence of a distinct genet. Both sites also exhibited some of the highest levels of clonal and genetic diversity. This suggests that Milford Haven harbours at least one distinct maerl population that is genetically diverse and geographically isolated from other populations in Britain. Interestingly, both maerl‐forming species showed similar patterns of differentiation where they coexist in sympatry. For instance, there was a strong population structure for maerl in Weymouth (and *L. corallioides* in Swanage; both sites in Dorset), which suggests geographical isolation from maerl in Cornwall. Additionally, at least one large clonal lineage in Cornwall was genetically differentiated in both species (e.g., *P. calcareum* in Gerrans Bay and *L. corallioides* in the Helford Estuary). Conversely, the Cornwall populations of *P. calcareum* were overall more admixed than *L. corallioides* . Given that a single maerl thallus is potentially over 100 years old (Foster 2001), and the dispersal capacity of maerl‐forming species is limited (Pardo et al. 2019), this suggests differing levels of historical or ongoing connectivity over long time‐scales. Moreover, in *P. calcareum* , shared ancestry between Cornwall populations and Zara Shoal in Northern Ireland, a distance of approximately 550 km, indicates a long and complex evolutionary history that has potentially been shaped by many factors, such as periodic changes in habitat suitability, connectivity and glacial refugia. Nevertheless, given low sample sizes in some sites, we may not have sampled all clonal lineages present, and therefore additional sampling of maerl would enhance inferences of clonality and population structure at these sites. Note, however, that due to the difficulty of identifying maerl to species level based on external morphology, especially in the field, this makes it challenging to design and carry out effective sampling strategies for specific maerl‐forming species, which in part explains why some sites in our study have lower sample sizes.

In a previous study, *P. calcareum* from St Mawes was found to be genetically distinct compared to all other maerl beds sampled in the north‐east Atlantic (Jenkins et al. 2021). In the current study, additional sampling of maerl beds in and around the Fal Estuary, and greater sequencing depth, has enabled further insight into this finding. We can now demonstrate that only the newly recognised coarse growth form of *P. calcareum* in St Mawes is genetically distinct. Evidence suggests this distinctiveness is due to triploidy, where one or more chromosomes have three copies rather than the two expected in maerl (Pardo et al. 2019). Triploidy in the *P. calcareum* form from the Fal Estuary was also postulated by Pardo and colleagues using microsatellite genotypes (Pardo et al. 2019), supporting our hypothesis that triploidy underlies the observed genetic differentiation in this coarse form of maerl. These findings raise two follow‐up questions: Is triploidy the result of hybridisation (allopolyploidy) or whole genome duplication (autopolyploidy)? And has triploidy contributed to the development of the markedly larger, bulkier growth form seen at this site, and, if so, is it a neutral or adaptive change? Given that no genomic data exist for other species in the *Phymatolithon* genus, and that no information is available on the karyology or physiology of this coarse form, further research will be needed to resolve these questions.

Our genomic analyses highlight several maerl populations that may be particularly at risk due to reduced clonal diversity, strong site‐specific clonality, or predicted high genomic offsets under future climate change. The coarse triploid *P. calcareum* population in St Mawes represents an Evolutionary Significant Unit (ESU) that is both genetically and morphologically distinct (Funk et al. 2012). It is also characterised by very low clonal diversity and extreme clonality, making it potentially vulnerable to environmental change and stochastic disturbance. The loss of such an ESU is equivalent to losing a distinct product of evolutionary history that has evolved unique and/or adaptive traits (Hoban et al. 2022). Similarly, Gerrans Bay harbours a single clonal lineage with strong population structuring, but no morphological differences were apparent in these maerl samples. East of Cornwall, the Weymouth maerl bed was highly differentiated from all other populations and displayed higher genomic offsets in climate projections, indicating increased risk of maladaptation under future environmental conditions. For *L. corallioides* , the Helford Estuary population also showed reduced clonal diversity and strong genetic differentiation, identifying it as another potentially vulnerable maerl bed.

These findings have direct relevance for conservation management because some of these maerl populations, including the most at‐risk sites, fall within existing Marine Conservation Zones (MCZs) or Special Areas of Conservation (SAC). For example, Weymouth and The Manacles are located within the Purbeck Coast MCZ and The Manacles MCZ, respectively, where maerl is listed as a protected feature. Likewise, St Mawes and the Helford Estuary are within the Fal and Helford SAC, Milford Haven lies within the Pembrokeshire Marine SAC, Zara Shoal is within the Strangford Lough SAC, and Illa de Ons is within the Espacio marino de las Rías Baixas de Galicia marine protected area. Some of these protected sites have legislation or byelaws in place to protect maerl, such as prohibiting scallop dredging and bottom towed gear (e.g., *The Manacles Marine Conservation Zone Fishing Restrictions Byelaw 2017*, Cornwall IFCA). Prohibiting these types of activities in all protected sites will be crucial for the preservation of maerl bed habitats since they are vulnerable to localised disturbance (Bernard et al. 2019; Hall‐Spencer 2000; Legrand et al. 2024) and have very slow recovery potential.

From a restoration and resilience perspective, populations with high clonal diversity, such as *L. corallioides* in St Austell Bay and Milford Haven, and *P. calcareum* at The Manacles and The Bizzies, may represent important reservoirs of genetic variation. These maerl beds could serve as priority sources of propagules for restoration or assisted gene flow, particularly if future disturbances reduce genetic diversity elsewhere. Conversely, populations dominated by a few clones, such as St Mawes (coarse triploid *P. calcareum* ) and the Helford Estuary ( *L. corallioides* ), may require a more precautionary management approach, with an increased emphasis on habitat protection rather than active translocation, given their limited recombination potential and uncertain adaptive capacity. However, while these genomic insights can inform restoration strategies, it is important to recognise that maerl is long‐lived and slow‐growing (Blake and Maggs 2003; Foster 2001), with very limited capacity for natural recovery once damaged. As such, maerl beds should be regarded as a functionally irreplaceable habitat, where proactive protection is more feasible and effective than restoration. Integrating genomic data into site‐specific management plans will therefore be crucial for designing conservation strategies that preserve both existing evolutionary lineages and the adaptive potential of maerl‐forming species across their range.

In conclusion, this study provides the first genomic resources for *P. calcareum* and *L. corallioides* and reveals differences in clonality, genetic diversity and population structure between species and within and across maerl beds in England and Wales. While extreme clonality was site‐specific in *P. calcareum* , with two genets producing many clones, *L. corallioides* generally displayed high clonal diversity. These contrasting patterns highlight differing adaptive potential and varying vulnerabilities to environmental change. Altogether, our results identified some maerl populations and lineages as evolutionarily distinct, while others retain high levels of genetic variation that may represent valuable reservoirs for resilience and restoration. These genomic insights, when integrated with ecological monitoring and experimental approaches, may inform efforts to prevent further loss or degradation of maerl beds and aid conservation interventions to recover or restore these irreplaceable, high natural capital value habitats.

## Conflicts of Interest

The authors declare no conflicts of interest.

## Supporting information


**Data S1:** cover_photo_maerl_Matt_Slater_Cornwall_Wildlife_Trust_2021

**Figure S1:** DNA extraction protocol

**Figure S2:** Maerl growth forms found in St Mawes, Falmouth.
**Figure S3:** Hamming genetic distances in P. calcareum (top) and L. corallioides (bottom).
**Figure S4:** The number of genets (multi‐locus lineages) and ramets (multi‐locus genotypes) is reported for Phymatolithon calcareum or Lithothamnion corallioides. For each species, clonality diversity and genetic diversity statistics for both genets and ramets are shown.
**Figure S5:** Phymatolithon calcareum PopCluster statistics (DLK2 and FSTIS).
**Figure S6:** Phymatolithon calcareum PopCluster results K2‐K8.
**Figure S7:** Phymatolithon calcareum principal component analysis (PCA).
**Figure S8:** Lithothamnion corallioides PopCluster statistics (DLK2 and FSTIS).
**Figure S9:** Lithothamnion corallioides PopCluster results K2‐K7.
**Figure S10:** Lithothamnion corallioides principal component analysis (PCA).
**Figure S11:** Phymatolithon calcareum genetic differentiation (FST).
**Figure S12:** Lithothamnion corallioides genetic differentiation (FST).
**Figure S13:** Phymatolithon calcareum genomic offsets.

**Table S1:** Information and references for the genomes used in the BUSCO analysis and phylogeny.
**Table S2:** Genome assembly statistics for coralline algal species available from the National Center for Biotechnology Information (NCBI) at the time of writing.

## Data Availability

Raw DNA sequence data and draft genome assemblies are available from the NCBI (BioProject: PRJNA682082; *Phymatolithon calcareum* assembly: SAMN41910763; *Lithothamnion corallioides* assembly: SAMN41910813). The modified BUSCO phylogenomics pipeline is available from GitHub: Tom‐Jenkins/BUSCO_phylogenomics. The custom Python scripts used for organelle annotation (extract_CDS.py) and for processing Kraken2 reports (process_kraken2_reports.py) are part of the Nextflow pipelines repository available from Zenodo: https://doi.org/10.5281/zenodo.14056754. R code and supporting data used in the analyses are also available from Zenodo: https://doi.org/10.5281/zenodo.12701452.
