# Supplementary figures and images for: Population Genomics Informs Resilience and Vulnerability of Habitat‐Building Coralline Algae
Source: Evol Appl. 2025 Nov 17;18(11):e70179. doi: 10.1111/eva.70179 (PMC12620667; doi:10.1111/eva.70179)

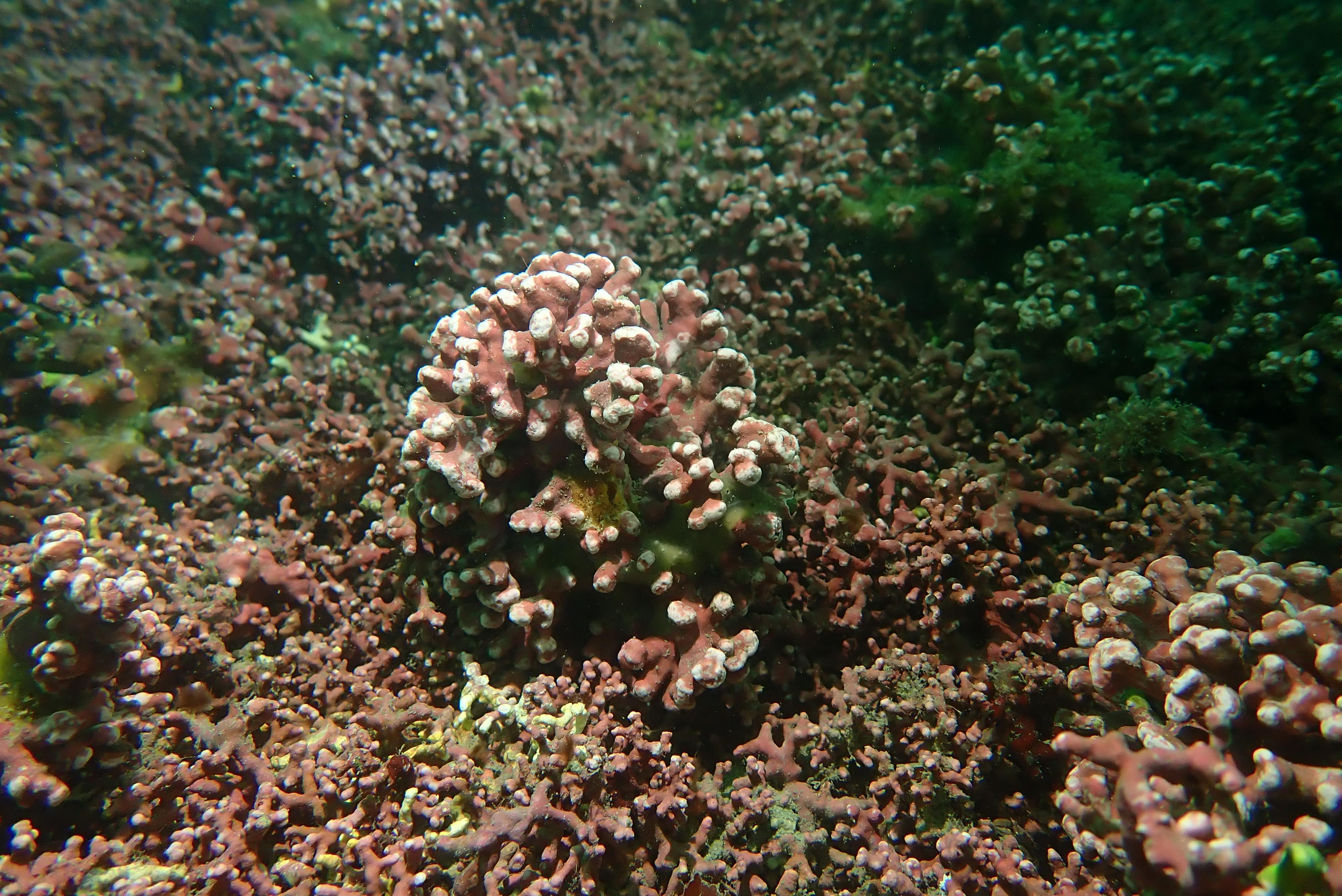

Supplement: Supplementary file 1 — Data S1: cover_photo_maerl_Matt_Slater_Cornwall_Wildlife_Trust_2021 [file EVA-18-e70179-s003.JPG]
