# Supplementary material for: Population Genomics Informs Resilience and Vulnerability of Habitat‐Building Coralline Algae
Source: Evol Appl. 2025 Nov 17;18(11):e70179. doi: 10.1111/eva.70179 (PMC12620667; doi:10.1111/eva.70179)
Supplement: Supplementary file 2 — Figure S1: DNA extraction protocol [file EVA-18-e70179-s001.docx]

**Supporting Information: Figure S1 DNA Extraction Protocol**

# Equipment / Reagents

- [DNeasy Blood & Tissue Kit](https://www.qiagen.com/us/products/discovery-and-translational-research/dna-rna-purification/dna-purification/genomic-dna/dneasy-blood-and-tissue-kit/) (Qiagen)
- [Proteinase K](https://www.neb.uk.com/products/neb-catalogue/protein-analysis,-exp-purification/proteinase-k,-molecular-biology-grade) (NEB)
- [RNase A](https://www.qiagen.com/us/products/discovery-and-translational-research/lab-essentials/enzymes/rnase-a/) (100 mg/ml)
- Buffer EB (Qiagen)
- 1.5ml or 2.0ml reaction tubes
- Vortexer
- Centrifuge
- Heat block or thermomixer
- Pestle and mortar

# Sample Preparation

- Remove live (pink) maerl rhodolith from ethanol and place on blue roll to remove excess ethanol. Briefly rinse with DI water.


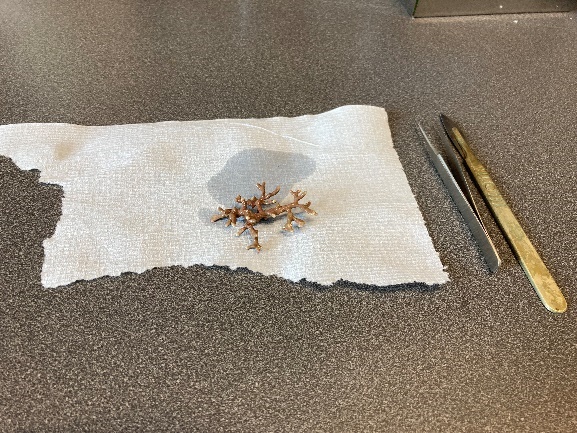


- Use a scalpel to remove debris, dirt and other non-target fauna/flora from rhodolith (if any).
- Place cleaned rhodolith in a pestle and mortar. Bash the rhodolith carefully into smaller pieces. Note: wash pestle and mortar with DI water and dry with blue roll in between samples.


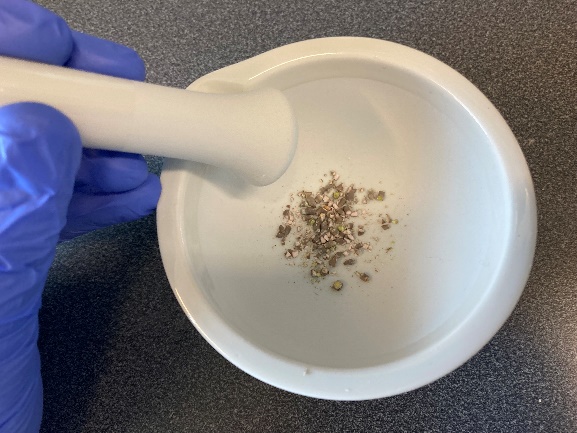


- Transfer only pieces where live maerl is evident to a 1.5ml tube. Repeat for all samples. Note: to increase DNA yield for each rhodolith, I have used two 1.5ml tubes per rhodolith sample and labelled them sample1a and sample1b. I keep 1a and 1b separate up until the spin column loading (step 5). Dispose of any maerl in the mortar not used.


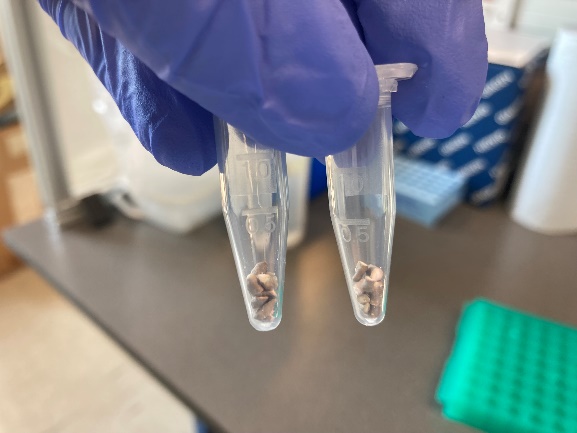

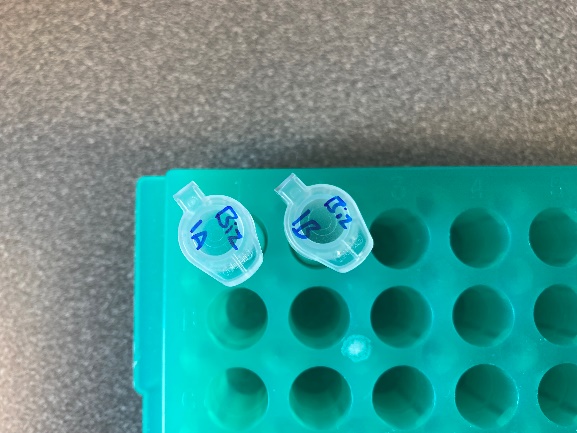


- For samples of encrusting red algae, a scalpel was used to detach bits off from rock which were then placed in a 1.5ml tube.


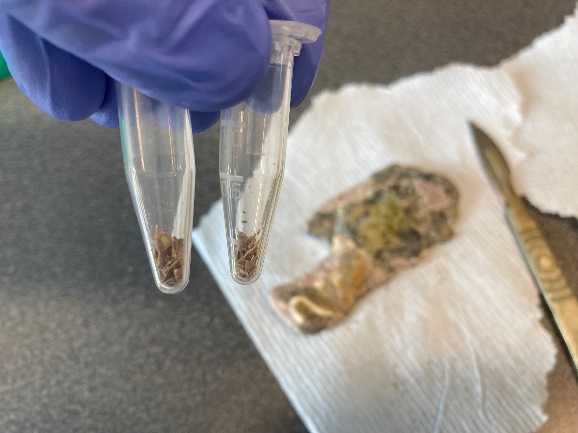


# Lysis

- Add 180ul buffer ATL to each 2ml tube. If sample is not fully submerged in buffer, add another 90ul buffer ATL (total volume: 270ul).


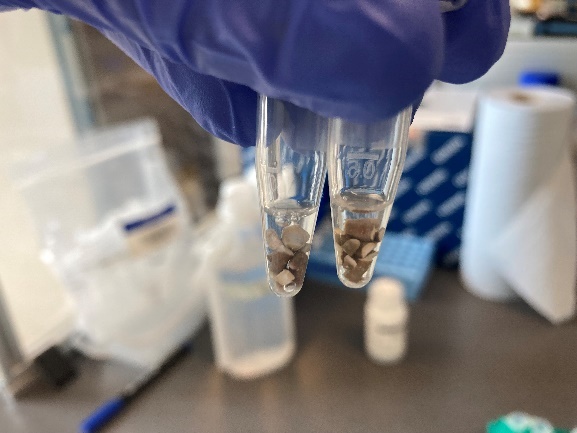


- Add 20ul proteinase K. If total volume is 270ul, add 30ul proteinase K.
- Vortex and pulse centrifuge tubes.
- Incubate at 56^o^C overnight (18-24 hours).

# Post-Lysis Processing

- Centrifuge tubes at 12,000xg for 2 minutes.
- Transfer as much supernatant as possible to a new 1.5ml tube.
- Add 4ul RNase A to each tube and incubate at 37^o^C for 30-60 minutes.
- Centrifuge tubes at 12,000xg for 5 minutes.
- Transfer supernatant to a new 1.5ml tube. Note: try to avoid the transfer of any pellets.

# Buffer AL & Ethanol

- Follow Qiagen protocol.

# Load Spin Columns

- Transfer to a silica membrane spin column. Note: if large white lysate particulates are present, vortex tube for 1-2 seconds prior to transfer to break up the lysate.
- Centrifuge at 8,000rpm for 1 minute.
- If you have two tubes for one sample (e.g. 1a and 1b), then repeat this step for each tube such that you end up with one spin column per sample. I.e. sample1a and sample1b now become just sample1.

# Washing

- Follow Qiagen protocol for washing with buffer AW1 and AW2.

# Elution

- Pipette 100ul buffer EB into the middle of each spin column. Note: buffer EB can be found in many other Qiagen kits, for example, the [PowerClean Cleanup Kit](https://www.qiagen.com/us/products/discovery-translational-research/dna-rna-purification/dna-purification/dna-clean-up/dneasy-powerclean-pro-cleanup-kit/). It is 10 mM Tris-Cl (pH 8.5) and contains no EDTA which is recommended because EDTA may inhibit subsequent enzymatic reactions.
- Incubate at room temperature for up to 4 minutes.
- Centrifuge at 8,000rpm for 1 minute.
