# Supplementary material for: Population Genomics Informs Resilience and Vulnerability of Habitat‐Building Coralline Algae
Source: Evol Appl. 2025 Nov 17;18(11):e70179. doi: 10.1111/eva.70179 (PMC12620667; doi:10.1111/eva.70179)
Supplement: Supplementary file 3 — Figure S2: Maerl growth forms found in St Mawes, Falmouth. Figure S3: Hamming genetic distances in P. calcareum (top) and L. corallioides (bottom). Figure S4: The number of genets (multi‐locus lineages) and ramets (multi‐locus genotypes) is reported for Phymatolithon calcareum or Lithothamnion corallioides. For each species, clonality diversity and genetic diversity statistics for both genets and ramets are shown. Figure S5: Phymatolithon calcareum PopCluster statistics (DLK2 and FSTIS). Figure S6: Phymatolithon calcareum PopCluster results K2‐K8. Figure S7: Phymatolithon calcareum principal component analysis (PCA). Figure S8: Lithothamnion corallioides PopCluster statistics (DLK2 and FSTIS). Figure S9: Lithothamnion corallioides PopCluster results K2‐K7. Figure S10: Lithothamnion corallioides principal component analysis (PCA). Figure S11: Phymatolithon calcareum genetic differentiation (FST). Figure S12: Lithothamnion corallioides genetic differentiation (FST). Figure S13: Phymatolithon calcareum genomic offsets. [file EVA-18-e70179-s004.docx]

**Harnessing population genomics to inform conservation of habitat-building coralline algae under climate change**

**Supporting Information**

Figure S2. Maerl growth forms found in St Mawes, Falmouth.


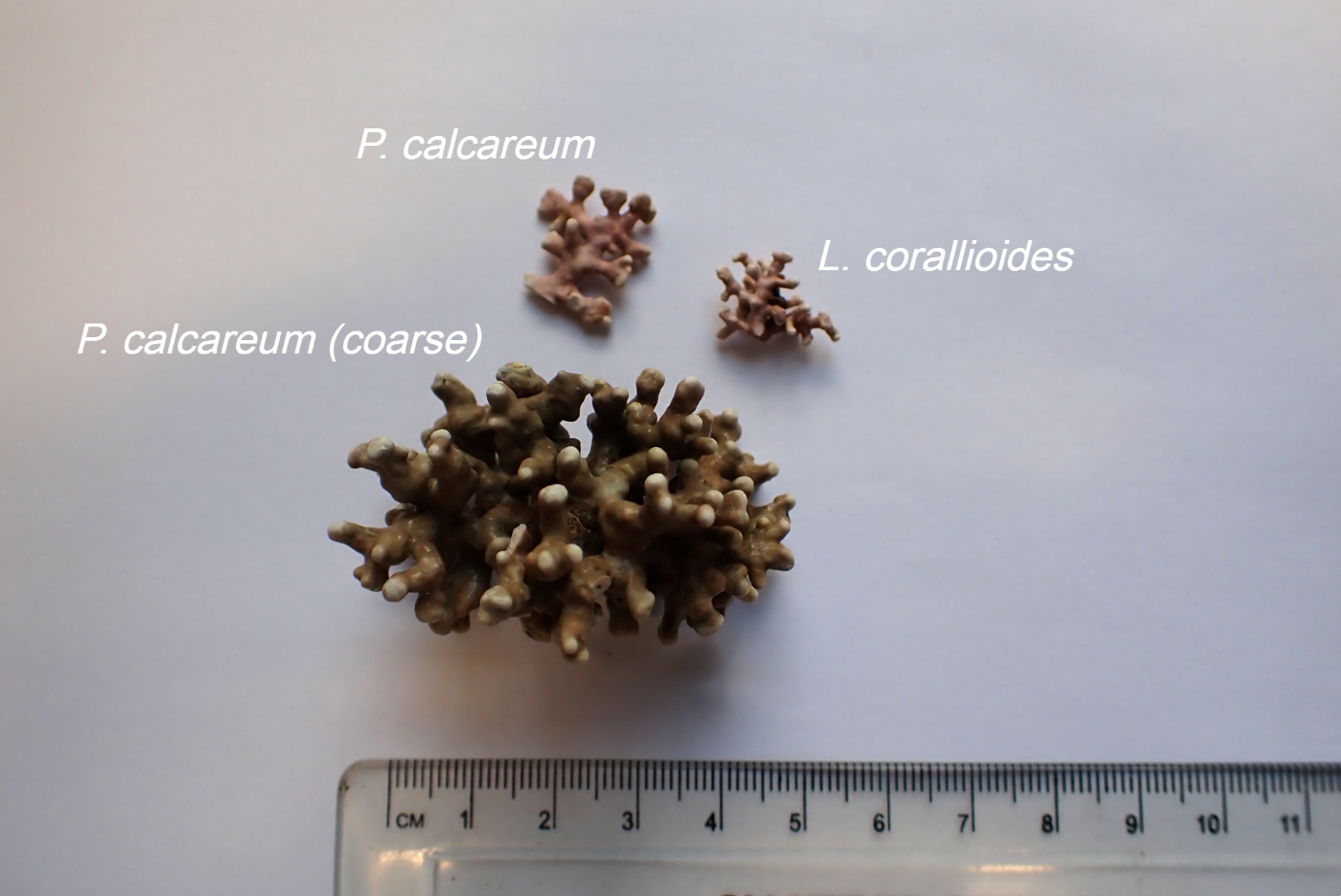


Figure S3. Hamming genetic distances in *P. calcareum* (top) and *L. corallioides* (bottom).


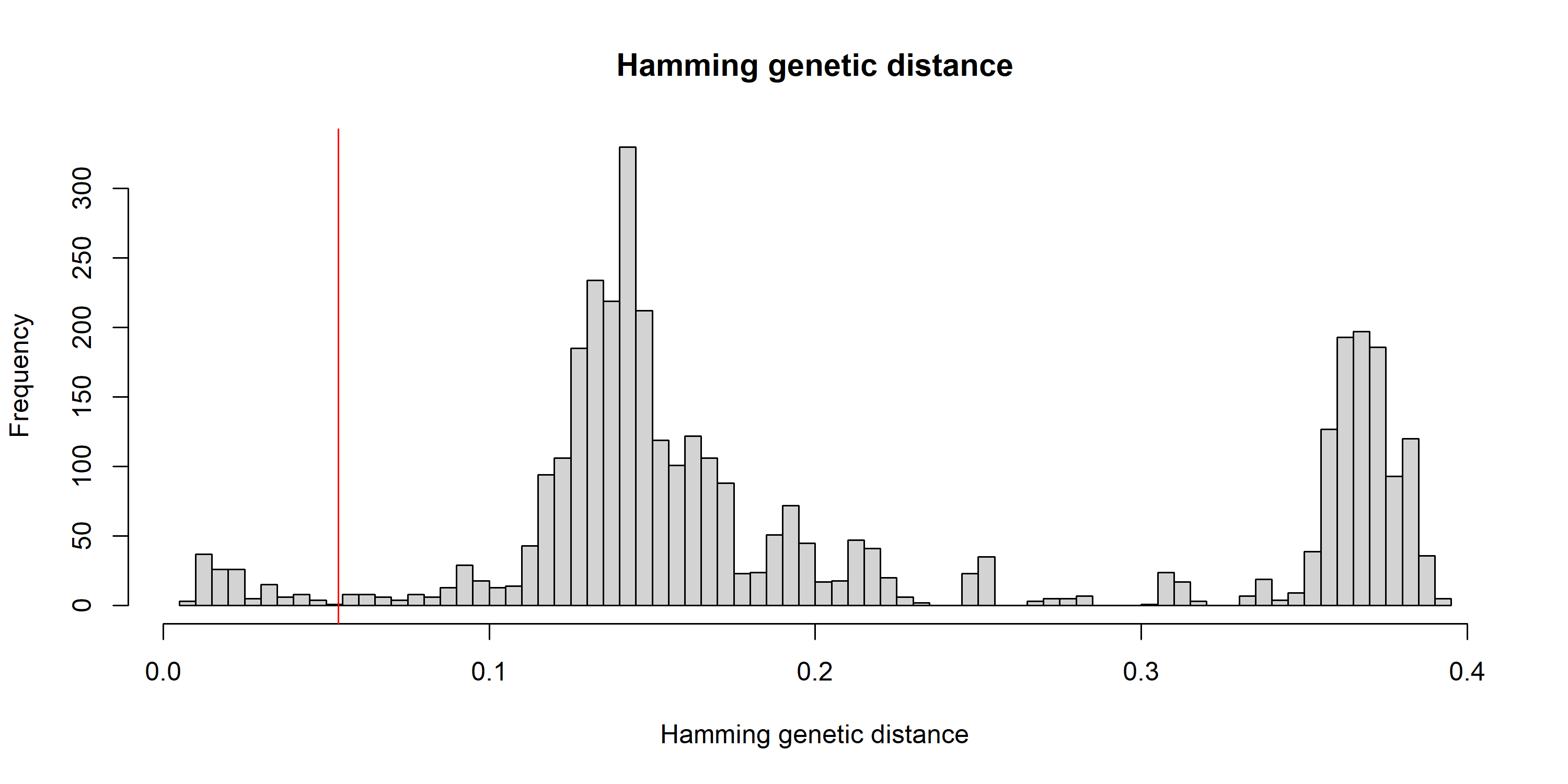

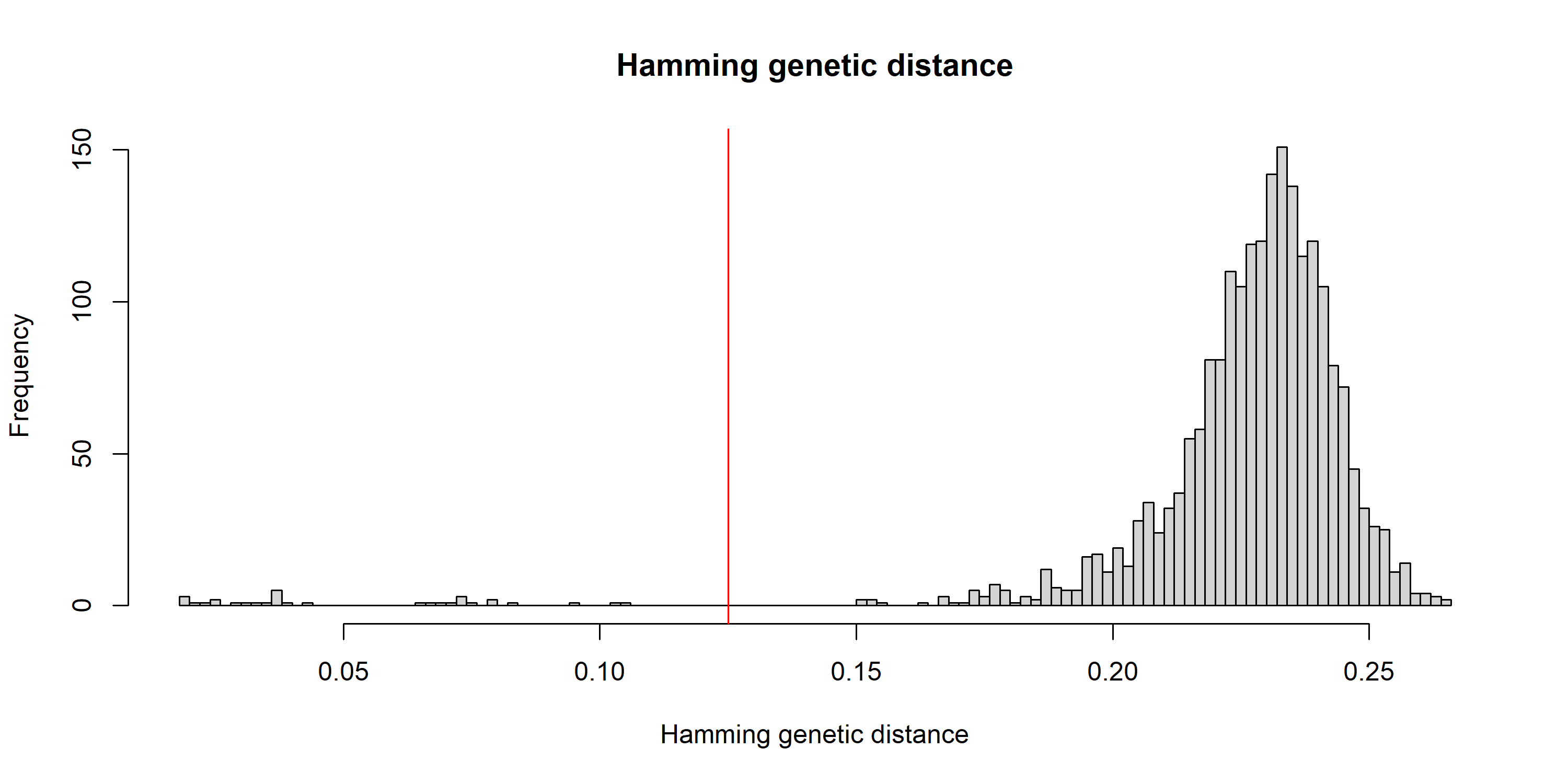


Figure S4. The number of genets (multi-locus lineages) and ramets (multi-locus genotypes) are reported for *Phymatolithon calcareum* or *Lithothamnion corallioides*. For each species, clonality diversity and genetic diversity statistics for both genets and ramets is shown.

| **Code** | **Genets (MLLs)** | **Ramets (MLGs)** | **Clonal diversity** | | **Genets diversity** | | | **Ramets diversity** | | |
| --- | --- | --- | --- | --- | --- | --- | --- | --- | --- | --- |
|  |  |  | **λ** | **Pareto β** | ***H*_O_** | ***H*_S_** | ***F*_IS_** | ***H*_O_** | ***H*_S_** | ***F*_IS_** |
|  |  |  |  |  |  |  |  |  |  |  |
| *Phymatolithon calcareum* |  |  |  |  |  |  |  |  |  |  |
| Aus | 7 | 10 | 0.73 | 1.78 | 0.27 | 0.23 | -0.18 | 0.28 | 0.23 | -0.22 |
| Biz | 8 | 15 | 0.77 | 2.00 | 0.26 | 0.21 | -0.20 | 0.26 | 0.21 | -0.22 |
| Maw | 6 | 9 | 0.71 | 1.63 | 0.31 | 0.22 | -0.34 | 0.31 | 0.22 | -0.35 |
| MawC | 3 | 15 | 0.22 | 0.43 | 0.61 | 0.34 | -0.78 | 0.65 | 0.34 | -0.85 |
| Ger | 6 | 15 | 0.56 | 0.86 | 0.30 | 0.22 | -0.30 | 0.28 | 0.20 | -0.33 |
| Man | 10 | 12 | 0.76 | 2.10 | 0.26 | 0.21 | -0.21 | 0.26 | 0.21 | -0.20 |
| Wey | 3 | 5 | 0.56 | 1.00 | 0.28 | 0.20 | -0.34 | 0.28 | 0.19 | -0.39 |
|  |  |  |  |  |  |  |  |  |  |  |
| *Lithothamnion corallioides* |  |  |  |  |  |  |  |  |  |  |
| Aus | 14 | 14 | 0.80 | *Inf* | 0.34 | 0.29 | -0.11 | 0.34 | 0.29 | -0.11 |
| Maw | 4 | 5 | 0.72 | 2.00 | 0.38 | 0.31 | -0.18 | 0.37 | 0.30 | -0.25 |
| Hel | 6 | 13 | 0.56 | 0.88 | 0.39 | 0.31 | -0.20 | 0.39 | 0.29 | -0.25 |
| Mil1 | 11 | 13 | 0.77 | 2.18 | 0.37 | 0.31 | -0.17 | 0.38 | 0.30 | -0.19 |
| Mil2 | 11 | 12 | 0.79 | 3.46 | 0.38 | 0.30 | -0.19 | 0.39 | 0.30 | -0.21 |

MLL, multi-locus lineage (proxy for the number of genets); Λ, Simpson’s diversity index; *Inf*, infinity denotes when Pareto β cannot be computed because no clones are present (the number of genets equals the number of ramets).

Figure S5. *Phymatolithon calcareum* PopCluster statistics (*D_LK_*_2_ and *F_STIS_*).


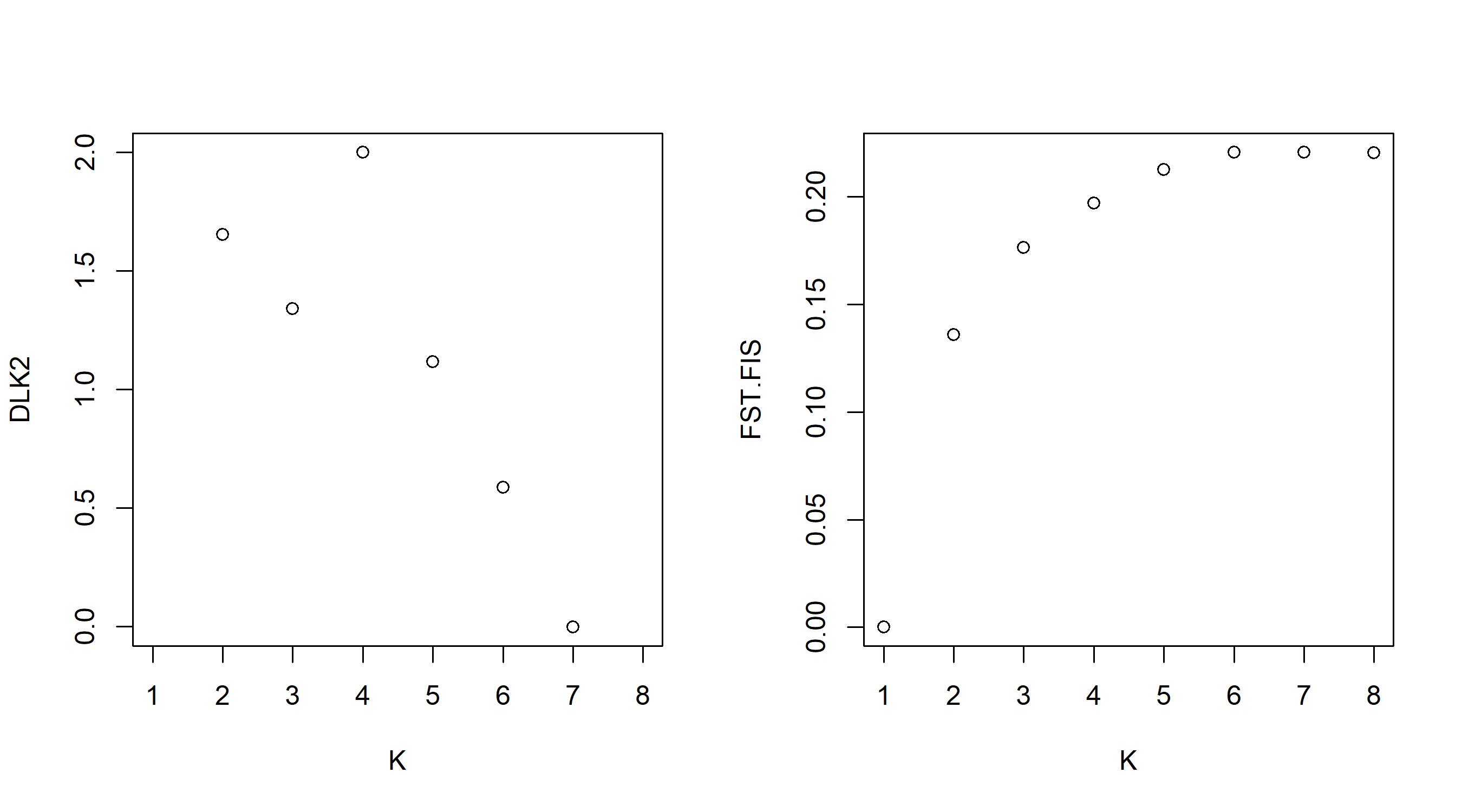


Figure S6. *Phymatolithon calcareum* PopCluster results *K*2-*K*8.


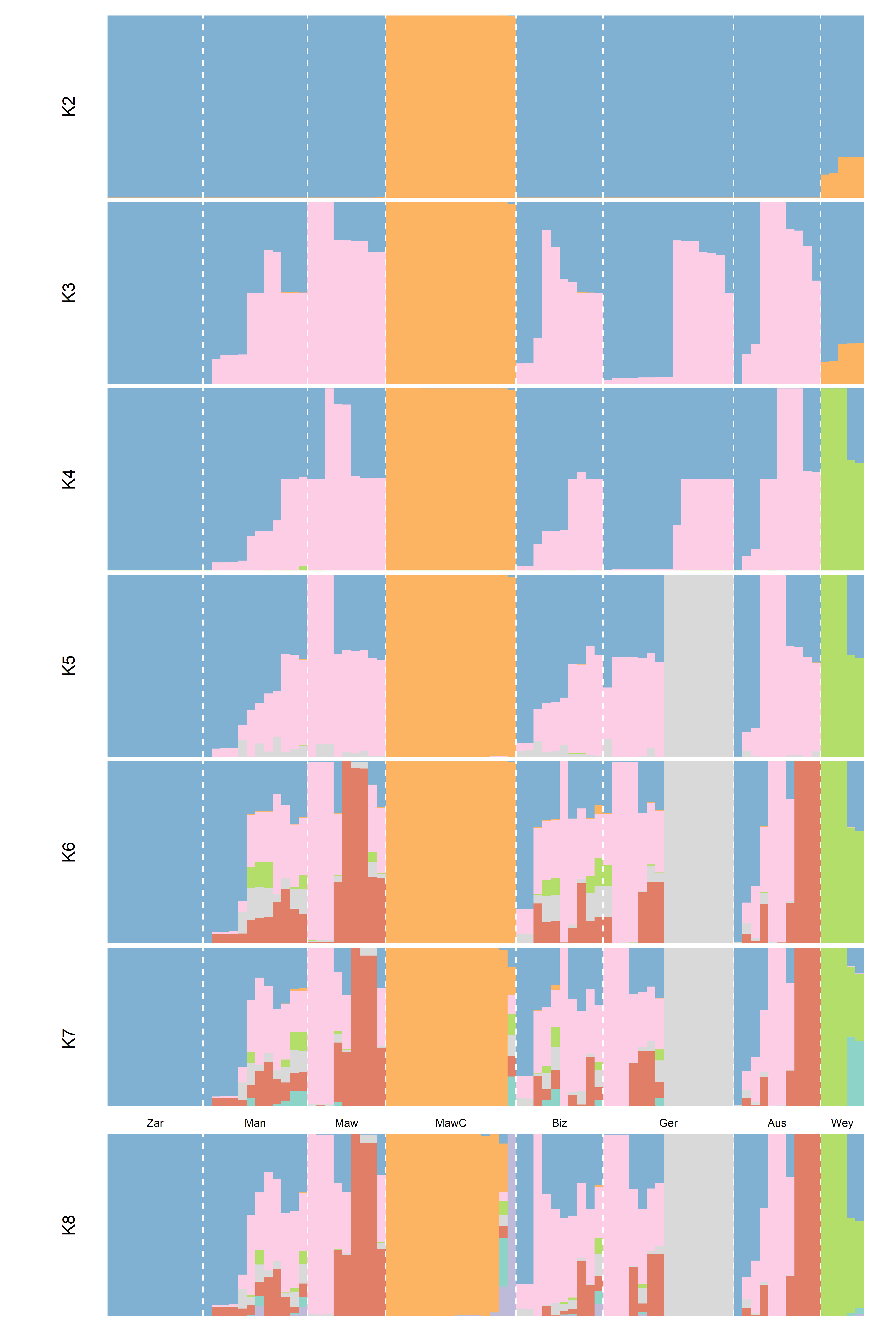


Figure S7. *Phymatolithon calcareum* principal component analysis (PCA).


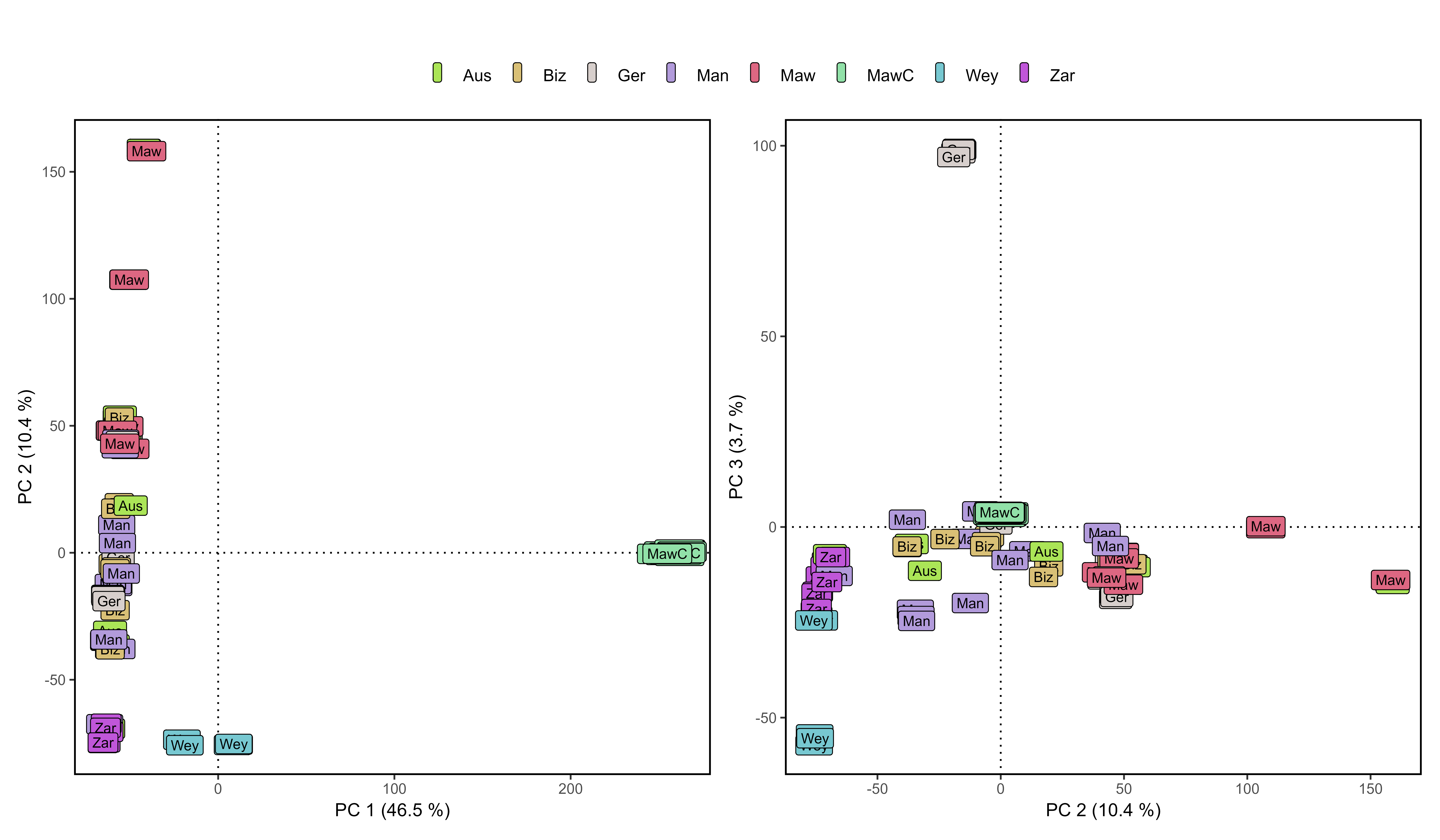


Figure S8. *Lithothamnion corallioides* PopCluster statistics (*D_LK_*_2_ and *F_STIS_*).


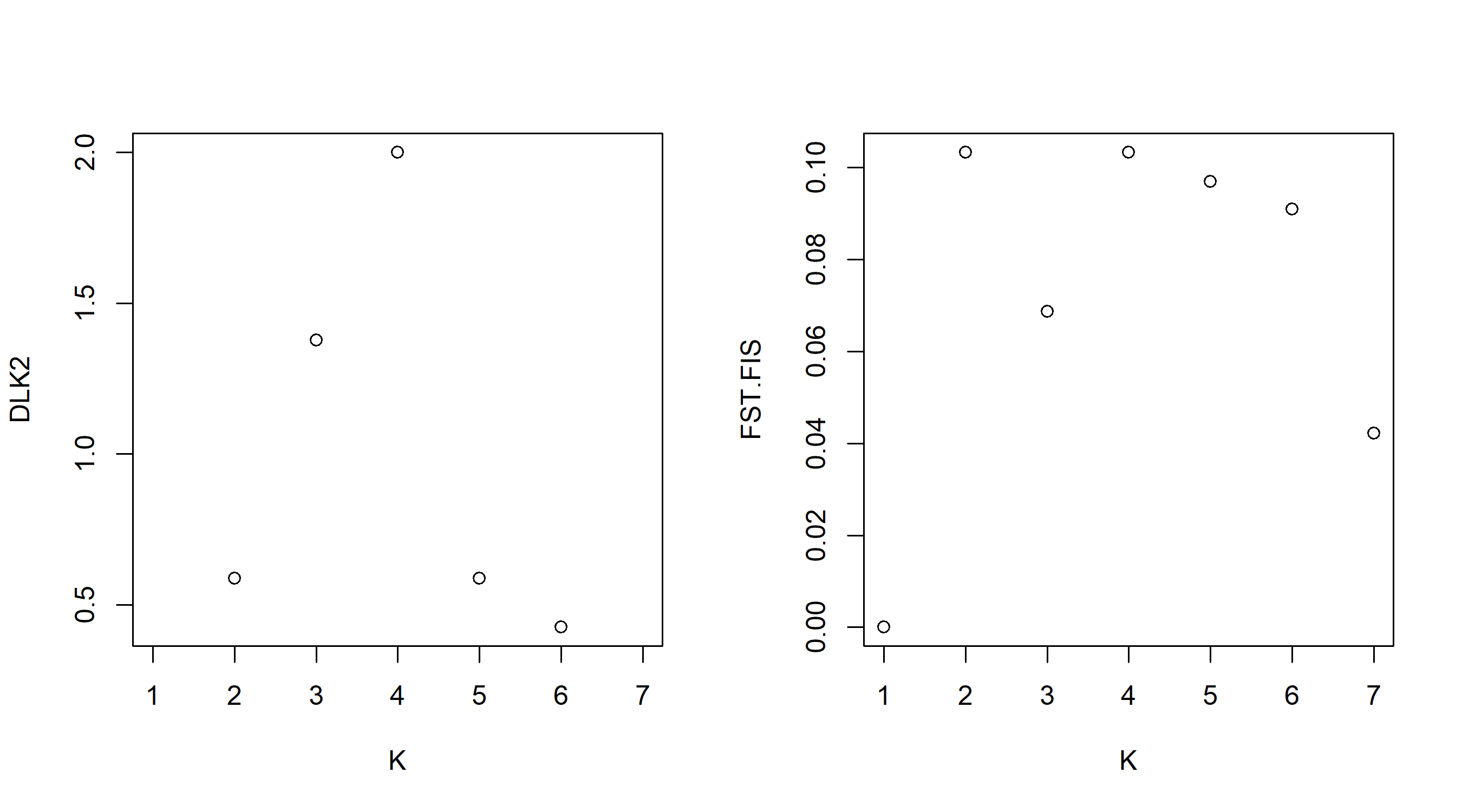


Figure S9. *Lithothamnion corallioides* PopCluster results *K*2-*K*7.


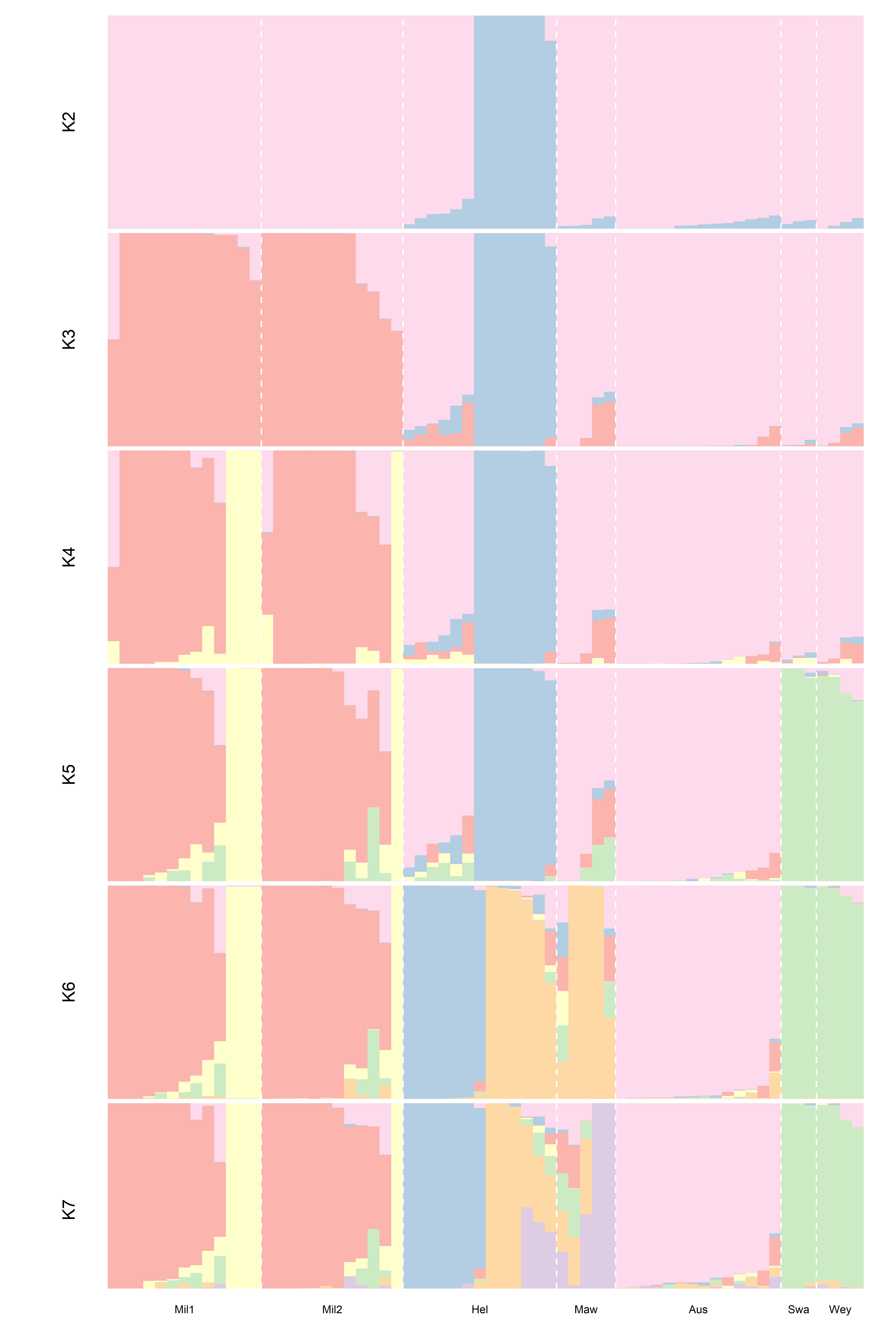


Figure S10. *Lithothamnion corallioides* principal component analysis (PCA).


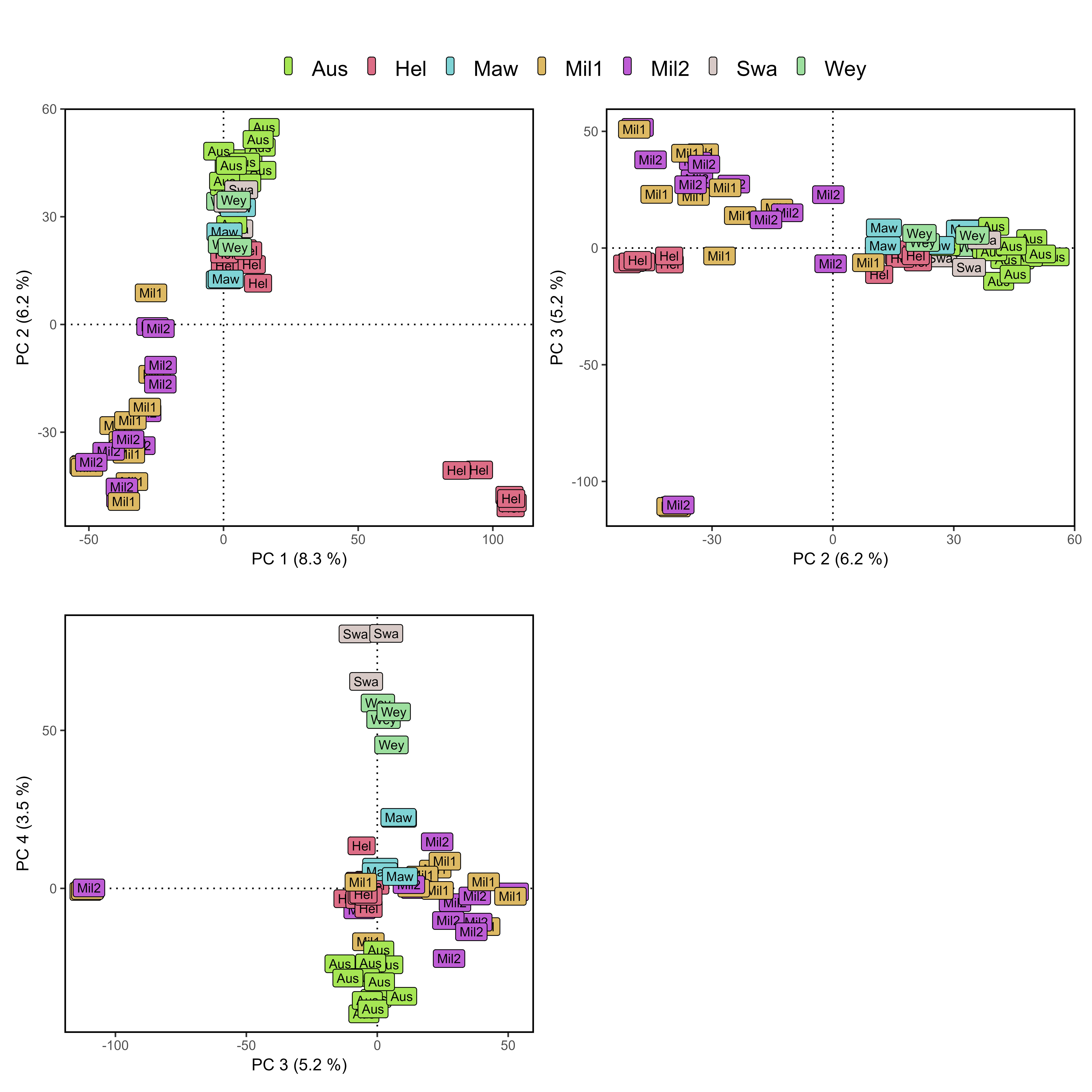


Figure S11. *Phymatolithon calcareum* genetic differentiation (*F*_ST_).

|  | Aus | Biz | Ger | Man | Maw | MawC |
| --- | --- | --- | --- | --- | --- | --- |
| Biz | 0.028 |  |  |  |  |  |
| Ger | 0.063 | 0.062 |  |  |  |  |
| Man | 0.046 | **0.019** | 0.060 |  |  |  |
| Maw | 0.015 | 0.071 | 0.099 | 0.087 |  |  |
| MawC | 0.331 | 0.351 | **0.373** | 0.359 | 0.346 |  |
| Wey | 0.219 | 0.191 | 0.223 | 0.171 | 0.266 | 0.333 |

Figure S12. *Lithothamnion corallioides* genetic differentiation (*F*_ST_).

|  | Aus | Hel | Maw | Mil1 | Mil2 | Swa |
| --- | --- | --- | --- | --- | --- | --- |
| Hel | 0.069 |  |  |  |  |  |
| Maw | 0.044 | 0.069 |  |  |  |  |
| Mil1 | 0.071 | 0.090 | 0.064 |  |  |  |
| Mil2 | 0.064 | 0.083 | 0.056 | **0.001** |  |  |
| Swa | 0.073 | **0.109** | 0.075 | 0.090 | 0.088 |  |
| Wey | 0.053 | 0.087 | 0.054 | 0.068 | 0.064 | 0.022 |

Figure S13. *Phymatolithon calcareum* genomic offsets.

| **Offset** | **Samples** |
| --- | --- |
| 0.034 | All 9 Trévignon samples. |
| 0.043 | All 11 Illa de Ons samples. |
| 0.045 | All 12 Bornalle samples. |
| 0.050 | All 11 Morlaix samples. |
| 0.054 | All 10 The Bizzies samples. |
| 0.056 | All 12 The Manacles samples. All 15 Gerrans Bay samples. |
| 0.057 | All 9 St Mawes samples. All 15 coarse St Mawes samples.  Maw22_04, Maw15_84, Maw22C_06, Maw11C_04, Maw22C_16, Maw11C_06, Maw15_82, Maw22_07, Maw11C_09, Maw11C_10, Maw11C_01, Maw11C_02, Maw22C_10, Maw11C_07, Maw22C_03, Maw15_27b, Maw22C_P11, Maw22_08, Maw22C_12, Maw15_77, Maw15_P1, Maw22C_14, Maw22C_15, Maw15_75 |
| 0.059 | All 10 St Austell samples. |
| 0.071 | All 5 Weymouth samples. |
| 0.073 | All 11 Zara Shoal samples. |
